# A systematic review of dissemination and implementation science capacity building programs around the globe

**DOI:** 10.1186/s43058-023-00405-7

**Published:** 2023-03-27

**Authors:** Clare Viglione, Nicole A. Stadnick, Beth Birenbaum, Olivia Fang, Julie A. Cakici, Gregory A. Aarons, Lauren Brookman-Frazee, Borsika A. Rabin

**Affiliations:** 1 UC San Diego Altman Clinical and Translational Research Institute Dissemination and Implementation Science Center, University of California San Diego, La Jolla, CA USA; 2 Department of Psychiatry, University of California San Diego, La Jolla, CA USA; 3 Child and Adolescent Services Research Center, San Diego, CA USA; 4 Herbert Wertheim School of Public Health and Human Longevity Science, University of California San Diego, La Jolla, CA USA; 5 School of Public Health, San Diego State University, San Diego, CA USA

**Keywords:** Dissemination and implementation science, Capacity building, Catalog, Inventory, Sustainment, International, Quality improvement, Knowledge translation, Education, Consultation, Training

## Abstract

**Background:**

Research centers and programs focused on dissemination and implementation science (DIS) training, mentorship, and capacity building have proliferated in recent years. There has yet to be a comprehensive inventory of DIS capacity building program (CBP) cataloging information about activities, infrastructure, and priorities as well as opportunities for shared resources, collaboration, and growth. The purpose of this systematic review is to provide the first inventory of DIS CBPs and describe their key features and offerings.

**Methods:**

We defined DIS CBPs as organizations or groups with an explicit focus on building practical knowledge and skills to conduct DIS for health promotion. CBPs were included if they had at least one capacity building activity other than educational coursework or training alone. A multi-method strategy was used to identify DIS CBPs. Data about the characteristics of DIS CBPs were abstracted from each program’s website. In addition, a survey instrument was developed and fielded to gather in-depth information about the structure, activities, and resources of each CBP.

**Results:**

In total, 165 DIS CBPs met our inclusion criteria and were included in the final CBP inventory. Of these, 68% are affiliated with a United States (US) institution and 32% are internationally based. There was one CBP identified in a low- and middle-income country (LMIC). Of the US-affiliated CBPs, 55% are embedded within a Clinical and Translational Science Award program. Eighty-seven CBPs (53%) responded to a follow-up survey. Of those who completed a survey, the majority used multiple DIS capacity building activities with the most popular being Training and Education (*n*=69, 79%) followed by Mentorship (*n*=58, 67%), provision of DIS Resources and Tools (*n*=57, 66%), Consultation (*n*=58, 67%), Professional Networking (*n*=54, 62%), Technical Assistance (*n*=46, 52%), and Grant Development Support (*n*=45, 52%).

**Conclusions:**

To our knowledge, this is the first study to catalog DIS programs and synthesize learnings into a set of priorities and sustainment strategies to support DIS capacity building efforts. There is a need for formal certification, accessible options for learners in LMICs, opportunities for practitioners, and opportunities for mid/later stage researchers. Similarly, harmonized measures of reporting and evaluation would facilitate targeted cross-program comparison and collaboration.

**Supplementary Information:**

The online version contains supplementary material available at 10.1186/s43058-023-00405-7.

Contributions to the literature

- There is rapid growth in dissemination and implementation science (DIS) interest, demand, investment, and opportunity, but there has yet to be a systematic review of DIS programs cataloging information about activities, infrastructure, and priorities
- We identify and describe existing DIS programs to build a comprehensive inventory of capacity building programs (CBPs) and activities
- We highlight areas for opportunity, collaboration, and growth to boost discrete DIS capacity building efforts across programs and allow for greater synergy and collaboration.

## Background

Interest in dissemination and implementation science (DIS) has grown exponentially over the last 15 years in the USA and internationally [1–3]. This growth can be attributed, in part, to increased investment from funding agencies. Of the 27 Institutes and Centers at the National Institutes of Health (NIH) in the USA, 18 participate in the Dissemination and Implementation Research in Health Program Announcement (PAR-18-017). The NIH Fogarty International Center has championed multiple “implementation science alliances” for specific initiatives such as preventing mother-to-child HIV transmission and adolescent HIV prevention and treatment [4, 5]. Other funders such as the Patient Centered Outcomes Research Institute and William T. Grant Foundation have specific RFAs requesting proposals that apply DIS methods [6, 7]. Clinical and Translational Science Award (CTSA) programs funded by the NIH’s National Center for Advancing Translational Sciences are now required to develop specialized programs to promote DIS as a prerequisite for funding (PAR-18-940). CTSAs require multidisciplinary capacity building services to guide researchers to effectively implement and disseminate evidence-based solutions, and for hubs to work together to tailor these solutions for different environments [8].

Capacity building is “a general term for a process of individual and institutional development which leads to higher levels of skills and greater ability to perform useful research” [2, 9]. DIS capacity building requires a multi-pronged approach where various types of activities of training, mentoring, technical assistance, pilot funding, learning collaboratives, tools, and other resources work synergistically together to support the development of skills across diverse DIS competencies (e.g., applying frameworks for multi-level evaluation, stakeholder analysis). Capacity building also entails the development of a group or collaborative of experts who regularly interact and engage in collaborations, peer mentoring, mentoring emerging and junior researchers, and idea generation, and collaborating on grant applications and publications. Examples of activities include targeted consultation, technical assistance for research teams, and the provision of educational materials and operational toolkits to guide researchers in systematically applying DIS. DIS capacity building programs across diverse institutions have been serving this role around the globe but may use different terms such as knowledge translation, knowledge exchange, and quality improvement to encompass DIS efforts.

Interest in and demand for DIS capacity building has also gained traction outside of the USA. Examples of international funding agencies for DIS research has expanded across global agencies like the World Health Organization, National Institute for Health and Care Research (UK), and United States Agency for International Development [10–12]. These DIS programs and opportunities also support networking and gathering for individuals passionate about DIS in global settings and provide a diverse set of activities and resources to support DIS capacity building.

To meet the interest and concomitant investment in DIS, several programs focused on DIS training, mentorship, and capacity building have been developed [2, 13]. Even with a multitude of DIS offerings, the demand for DIS training and mentoring far exceeds available opportunities. For instance, the NIH-funded Training Institute for Dissemination and Implementation Research in Cancer (TIDIRC) program received 266 applicants for 35 slots in its first year and the NIH, NIDA, and VA-funded Implementation Research Institute routinely receives five applications for every one fellowship slot [14]. As such, finding ways to accelerate the pace of DIS capacity building and training has been recognized as an international priority, with experts and organizations (e.g., the NIH) concluding that we need greater access to capacity building for all levels of DIS researchers and practitioners [15].

Despite the rapid growth in DIS interest, demand, investment, and opportunity, to our knowledge there has yet to be a systematic review of DIS programs cataloging information about activities, infrastructure, and priorities [2]. Davis & D’Lima reviewed academic literature to catalogue teaching and training initiatives in DIS ranging from short training courses to intensive institutes. Our work expands on their review by systematically searching the internet for DIS capacity building programs inclusive of but not limited to training institutes. Also, because of the diversity of DIS research and practice areas (e.g., cancer, infectious disease, behavioral and school health) and the often-siloed nature of distinct academic disciplines, there is a need to examine areas of overlap and redundancy across DIS programs as well as explore opportunities for shared resources, collaboration, and growth [16]. We acknowledge and build on the work of Darnell and colleagues (2017) that reviewed and characterized existing DIS resource initiatives and identified multiple and overlapping “non-interactive” DIS resources (e.g., resource libraries, archived talks) [16]. In this systematic review, we identify and describe existing DIS infrastructure to build a comprehensive inventory of capacity building programs and activities. We highlight areas for opportunity, collaboration, and growth to boost discrete DIS capacity building efforts across programs and allow for greater synergy and collaboration.

## Methods

**Fig. 1:**
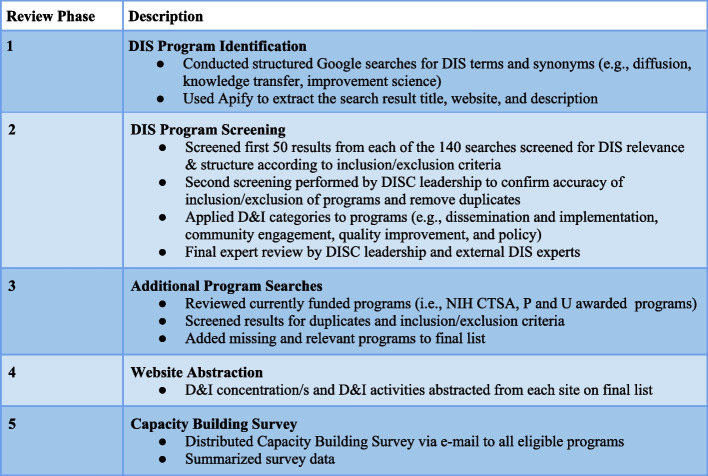
Systematic review phases

We used a multi-method strategy to identify DIS capacity building programs (from here on referred to as DIS CBPs). The phases of our search, review, survey, data collection, and synthesis process are summarized below and in Fig. 1. The search followed the Preferred Reporting Items of Systematic Reviews and Meta-analyses (PRISMA) guidelines (see Additional File with a PRISMA checklist). The search commenced in August 2020 and the review finished in January 2022. The study was reviewed for approval by the UC San Diego Health Sciences Institutional Review Board (T28074).

### Phase 1—DIS capacity building program search strategy

To identify all DIS CBPs, we used Google searches for “dissemination and implementation” and nine synonyms that are common outside of the USA (e.g., diffusion, knowledge transfer, improvement science) with and without the word “program” along with 13 synonyms (e.g., institute, center, collaborative) [17]. See Table 1 for a full list of search terms with the corresponding results. The first 50 search results were extracted from each unique search. Due to known shortcomings that result in an inability to reproduce Google search results for systematic reviews, we used Apify, an online web-scraping tool, that allowed us to automatically extract the search result title, website, and description thus limiting the number of searches run [18, 19]. We also crosschecked the DIS Program Search Strategy by confirming inclusion of a starter list of DIS CBPs that was created by the coauthors of this paper based on their expert knowledge.

**Table 1:** Dissemination and implementation science capacity building program

***Google search results***	
**Dissemination and implementation**	**Search results**
"dissemination and implementation" AND "center"	1,730,000
"dissemination and implementation" AND "centre"	1,630,000
"dissemination and implementation" AND "institute"	1,660,000
"dissemination and implementation" AND "institution"	1,350,000
"dissemination and implementation" AND "program"	2,390,000
"dissemination and implementation" AND "programme"	1,430,000
"dissemination and implementation" AND "collaborative"	765,000
"dissemination and implementation" AND "consortium"	80
"dissemination and implementation" AND "team"	1,610,000
"dissemination and implementation" AND "alliance"	1,130,000
"dissemination and implementation" AND "core"	1,310,000
"dissemination and implementation" AND "division"	94
"dissemination and implementation" AND "group"	3,400,000
"dissemination and implementation" AND "department"	2,320,000
**Total**	20,725,174
**Dissemination**	**Search results**
"dissemination " AND "center"	89,700,000
"dissemination" AND "centre"	86,200,000
"dissemination" AND "institute"	66,200,000
"dissemination" AND "institution"	74,200,000
"dissemination" AND "program"	61,300,000
"dissemination" AND "programme"	110,000,000
"dissemination" AND "collaborative"	53,100,000
"dissemination" AND "consortium"	60,900,000
"dissemination" AND "team"	85,600,000
"dissemination" AND "alliance"	26,800,000
"dissemination" AND "core"	77,500,000
"dissemination" AND "division"	81,700,000
"dissemination" AND "group"	119,000,000
"dissemination" AND "department"	104,000,000
**Total**	1,096,200,000
**Implementation**	**Search results**
"implementation" AND "center"	625,000,000
"implementation" AND "centre"	707,000,000
"implementation" AND "institute"	447,000,000
"implementation" AND "institution"	341,000,000
"implementation" AND "program"	763,000,000
"implementation" AND "programme"	357,000,000
"implementation" AND "collaborative"	179,000,000
"implementation" AND "consortium"	83,100,000
"implementation" AND "team"	557,000,000
"implementation" AND "alliance"	127,000,000
"implementation" AND "core"	519,000,000
"implementation" AND "division"	481,000,000
"implementation" AND "group"	559,000,000
"implementation" AND "department"	606,000,000
**Total**	6,351,100,000
**Diffusion**	**Search results**
"diffusion" AND "center"	290,000,000
"diffusion" AND "centre"	361,000,000
"diffusion" AND "institute"	270,000,000
"diffusion" AND "institution"	151,000,000
"diffusion" AND "program"	213,000,000
"diffusion" AND "programme"	309,000,000
"diffusion" AND "collaborative"	52,500,000
"diffusion" AND "consortium"	25,500,000
"diffusion" AND "team"	118,000,000
"diffusion" AND "alliance"	111,000,000
"diffusion" AND "core"	95
"diffusion" AND "division"	147,000,000
"diffusion" AND "group"	384,000,000
"diffusion" AND "department"	246,000,000
**Total**	2,678,000,095
**Knowledge Transfer**	**Search results**
"knowledge transfer" AND "center"	25,600,000
"knowledge transfer" AND "centre"	26,400,000
"knowledge transfer" AND "institute"	8,460,000
"knowledge transfer" AND "institution"	3,980,000
"knowledge transfer" AND "program"	7,300,000
"knowledge transfer" AND "programme"	4,590,000
"knowledge transfer" AND "collaborative"	5,180,000
"knowledge transfer" AND "consortium"	1,720,000
"knowledge transfer" AND "team"	26,100,000
"knowledge transfer" AND "alliance"	2,390,000
"knowledge transfer" AND "core"	8,240,000
"knowledge transfer" AND "division"	3,830,000
"knowledge transfer" AND "group"	13,900,000
"knowledge transfer" AND "department"	9,300,000
**Total**	146,990,000
**Knowledge Translation**	**Search results**
"knowledge translation" AND "center"	605,000
"knowledge translation" AND "centre"	999,000
"knowledge translation" AND "institute"	711,000
"knowledge translation" AND "institution"	934,000
"knowledge translation" AND "program"	584,000
"knowledge translation" AND "programme"	258,000
"knowledge translation" AND "collaborative"	1,080,000
"knowledge translation" AND "consortium"	642,000
"knowledge translation" AND "team"	673,000
"knowledge translation" AND "alliance"	823,000
"knowledge translation" AND "core"	1,170,000
"knowledge translation" AND "division"	1,040,000
"knowledge translation" AND "group"	638,000
"knowledge translation" AND "department"	657,000
**Total**	10,814,000
**Improvement Science**	**Search results**
"improvement science" AND "center"	118,000
"improvement science" AND "centre"	285,000
"improvement science" AND "institute"	133,000
"improvement science" AND "institution"	430,000
"improvement science" AND "program"	127,000
"improvement science" AND "programme"	102,000
"improvement science" AND "collaborative"	467,000
"improvement science" AND "consortium"	196,000
"improvement science" AND "team"	138,000
"improvement science" AND "alliance"	402,000
"improvement science" AND "core"	99,800
"improvement science" AND "division"	464,000
"improvement science" AND "group"	621,000
"improvement science" AND "department"	131,000
**Total**	3,713,800
**Knowledge Exchange**	**Search results**
"knowledge exchange" AND "center"	3,280,000
"knowledge exchange" AND "centre"	5,080,000
"knowledge exchange" AND "institute"	3,900,000
"knowledge exchange" AND "institution"	1,510,000
"knowledge exchange" AND "program"	2,850,000
"knowledge exchange" AND "programme"	3,260,000
"knowledge exchange" AND "collaborative"	2,460,000
"knowledge exchange" AND "consortium"	951,000
"knowledge exchange" AND "team"	4,930,000
"knowledge exchange" AND "alliance"	1,380,000
"knowledge exchange" AND "core"	2,990,000
"knowledge exchange" AND "division"	1,540,000
"knowledge exchange" AND "group"	6,360,000
"knowledge exchange" AND "department"	3,970,000
**Total**	44,461,000
**Quality Improvement**	**Search results**
"quality improvement" AND "center"	30,300,000
"quality improvement" AND "centre"	21,000,000
"quality improvement" AND "institute"	30,200,000
"quality improvement" AND "institution"	11,500,000
"quality improvement" AND "program"	23,500,000
"quality improvement" AND "programme"	16,800,000
"quality improvement" AND "collaborative"	13,200,000
"quality improvement" AND "consortium"	2,390,000
"quality improvement" AND "team"	21,600,000
"quality improvement" AND "alliance"	4,690,000
"quality improvement" AND "core"	20,800,000
"quality improvement" AND "division"	15,800,000
"quality improvement" AND "group"	19,900,000
"quality improvement" AND "department"	29,200,000
**Total**	260,880,000
**Community Engagement**	**Search results**
"community engagement" AND "center"	20,700,000
"community engagement" AND "centre"	26,200,000
"community engagement" AND "institute"	16,600,000
"community engagement" AND "institution"	8,360,000
"community engagement" AND "program"	24,100,000
"community engagement" AND "programme"	9,520,000
"community engagement" AND "collaborative"	8,410,000
"community engagement" AND "consortium"	2,230,000
"community engagement" AND "team"	22,300,000
"community engagement" AND "alliance"	7,050,000
"community engagement" AND "core"	23,400,000
"community engagement" AND "division"	18,200,000
"community engagement" AND "group"	22,000,000
"community engagement" AND "department"	18,400,000
**Total**	227,470,000
**Overall total**	**10,840,354,069**

### Phase 2—DIS program website screening using inclusion criteria

A three-tiered screening process was used to identify the initial list of DIS CBPs. First, trained research assistants screened resultant webpages from Google (*n*=7000) to identify DIS programs meeting eligibility criteria. CBPs were defined as an entity (e.g., organization, program or center) with at least one capacity building activity (e.g., consultation, technical assistance, networking events, journal club meetings) with an explicit focus or goal of building practical knowledge and skills to conduct DIS for public or population health work. CBPs affiliated with academic organizations including Clinical and Translational Science Award Program, governmental and funding entities, the United States Veterans Health Administration, or not for profits or collaboratives focusing on education, business, or technology with a stated emphasis on public or population health were also included.

Programs were included if (a) the focus was on DIS or one of the search terms listed in Table 1 (e.g., knowledge translation); (b) the organization offered at least one capacity building activity other than coursework, training, or a static educational material (e.g., an implementation science resource guide) as our intention was to identify CBPs that provide more comprehensive, multi-component capacity building activities rather than focusing solely on training or education; and (c) the program’s focus was in service of public or population health. All programs that were provisionally included were discussed to verify they were classified appropriately according to these criteria.

Second, as part of quality assessment, the study coordinator independently reviewed all programs in depth to confirm eligibility and screened for duplicate programs. The review team (who included a study coordinator, research assistants, a doctoral student, and faculty researchers) met biweekly to refine inclusion and exclusion criteria and come to consensus about specific programs. Lastly, two faculty researcher team members independently reviewed 50% of the included programs to confirm program inclusion. Each faculty, researcher team member then checked each other’s decisions on their respective 50%, with a select number tagged for full team discussion.

### Phase 3—Targeted searches for additional DIS programs

To validate the initial list of programs and ensure an exhaustive list was compiled, we consulted 6 experts in the DIS field to identify missing programs. The DIS experts consulted were nationally and internationally recognized DIS researchers who were connected to our research team or identified via snowball sampling. Experts represented multiple perspectives including academic researchers with international reputation in DIS, government funders, and DIS experts with experience leading DIS training and capacity building. In addition to DIS expert nomination of missing programs, searches were conducted via NIH Reporter. This consisted of targeted searches on 2020 funding awards for DIS programs funded by NIH with known DIS components including CTSA and multi-project research applications (e.g., U01, P50s). Results were screened for duplicates and eligibility.

### Phase 4—Website abstraction for all programs and data cleaning

The full website for each CBP was reviewed for capacity building information and activities. Information was abstracted by research assistants using an iteratively developed abstraction form. Abstracted information was reviewed by BR and NAS after decisions about program inclusion were made following the robust reliability assessments described in Quality Assurance section below. Fields for abstraction included locational information, primary website, DIS concentration, and DIS capacity building activities (see Table 2). All identified programs were categorized with uniform descriptors to characterize their DIS focus and capacity building activities. Table 3 describes the agreed upon definitions for the capacity building activities. In addition, we contacted 5 academic institutions each with multiple DIS programs to clarify whether to list their programs separately or to include them as a single institutional center. All indicated to include each program individually.

**Table 2:** Results from systematic review of DIS programs

Host institution	Program name	Country / state / province	Website	D&I concentration Dissemination & Implementation (DI) Quality Improvement (QI) Knowledge Translation (KT) ***Subspecialty****:* *Community Engagement (CE)* *Policy*	Capacity building activities Conferences/Workshops Consultation Data Analysis Database Fellowships Framework/Tool Development Funding Guideline Dev. Internships Mentorship Research Seminars/Webinars Training/Courses Training Materials Video Channel Work Placement
Survey respondents (*n*= 87)					
ABCT Dissemination and Implementation Science Special Interest Group (DIS SIG)	ABCT Dissemination and Implementation Science Special Interest Group (DIS SIG)	USA	https://dissig.tidyhq.com/	DI	Conferences/Workshops Mentorship Training Materials
Boston University	Clinical and Translational Science Institute (CTSI)	USA / Boston	https://www.bu.edu/ctsi/	DI	Conferences/Workshops Consultation Framework/Tool Development Funding Mentorship Research Seminars/Webinars Training/Courses
Boston University	Evans Center for Implementation and Improvement Sciences (CIIS)	USA / Boston	https://sites.bu.edu/ciis/	DI	Conferences/Workshops Consultation Data Analysis Fellowships Research
Bradford Teaching Hospitals NHS FT/Bradford Institute for Health Research	Improvement Science theme of the Yorkshire and Humber Applied Research Collaboration	United Kingdom	https://www.arc-yh.nihr.ac.uk/what-we-do/improvement-science	DI, QI	Conferences/Workshops Research Training/Courses Video Channel
Brown University	Brown Implementation Science Core, Department of Psychiatry and Human Behavior	USA / Rhode Island	https://sites.google.com/brown.edu/implementation-science-core/home	DI	Conferences/Workshop Framework/Tool Development Seminars/Webinars
Campbell Collaboration	Knowledge Translation and Implementation Coordinating Group at Campbell Collaboration	Norway	https://www.campbellcollaboration.org/contact/coordinating-groups/knowledge-translation-and-implementation.html#	KT, DI	Guideline Dev. Mentorship Research Training/Courses
Canadian Knowledge Transfer and Exchange Community of Practice (KTECOP)	Canadian Knowledge Transfer and Exchange Community of Practice (KTECOP)	Canada	https://www.ktecop.ca/	KT	Conferences/Workshops Seminars/Webinars Training/Courses
Columbia University	Implementation Science Initiative with Irving Institute CTSA at Columbia University (CUIMC)	USA / New York	https://www.irvinginstitute.columbia.edu/implementation-science	DI	Conferences/Workshops Consultation Funding Research Seminars/Webinars Training Materials
Diabetes Action Canada	Knowledge Transfer Program	Canada / Ontario	https://diabetesaction.ca/our-projects-commitments-and-progress/	KT	Framework/Tool Development Funding Mentorship Research Training/Courses
Emory University	The Intervention Development, Dissemination, and Implementation Developing Shared Resource, Winship Cancer Institute	USA / Georgia	https://winshipcancer.emory.edu/research/shared-resources/intervention-development-dissemination-implementation.html	DI	Consultation Research
European Implementation Collective	European Implementation Collective (EIC)	Europe	https://implementation.eu/	DI	Database Training Materials
Gambling Research Exchange Ontario (Greo)	Gambling Research Exchange Ontario (Greo)	Canada / Ontario	www.greo.ca	KT	Consultation Data Analysis Database Funding Guideline Development Research Seminars/Webinars Training Materials
Harvard Medical School	Harvard Catalyst/Community Engagement Program	USA / Massachusetts	https://catalyst.harvard.edu/	DI	Consultation Database Fellowships Framework/Tool Development Mentorship Seminars/Webinars Training/Courses
Institute for Healthcare Improvement (IHI)	Institute for Healthcare Improvement (IHI)	USA / Massachusetts	www.ihi.org	QI	Conferences/Workshops Consultation Fellowships Seminars/Webinars Training/Courses
Institute for Knowledge Translation (iIKT) Academy	Institute for Knowledge Translation (iKT) Academy	USA / Indiana	https://www.knowledgetranslation.org/academy-2	KT, QI	Consultation Mentorship Training/Courses
Indiana University School of Medicine	Center for Health Innovation and Implementation Science	USA / Indiana	https://indianactsi.org/	DI	Data Analysis Fellowships Framework/Tool Development Funding Internships Mentorship Research Seminars/Webinars Training/Courses Video Channel
Intermountain Healthcare Delivery Institute	Care Delivery Science	USA / Utah	https://intermountainhealthcare.org/about/transforming-healthcare/hdi/care-delivery-science/	DI, QI	Fellowships Internships Research Training/Courses
Johns Hopkins University	Inter-CFAR Fellowship in Implementation Science & HIV	USA / Maryland	https://hopkinscfar.org/science-cores/implementation-science-swg/	DI	Consultation Fellowships
Kaiser Permanente	Care Improvement Research Team (CIRT)	USA / California	https://www.kp-scalresearch.org/division/health-services-research-implementation-science/	DI, QI	Fellowships Research
King's College London	Centre for Implementation Science	United Kingdom	https://www.kcl.ac.uk/research/cis	DI, QI	Guideline Dev. Research
McMaster University	The National Collaborating Centre for Methods and Tools	Canada	https://www.nccmt.ca/about/vision-mission-goals	KT	Conferences/Workshops Framework/Tool Development Mentorship Training/Courses Training Materials
Michael Smith Foundation for Health Research	Implementation Science Teams	Canada / British Columbia	https://www.msfhr.org/kt-at-msfhr	KT	Framework/Tool Development Funding Seminars/Webinars Training Materials
National Cancer Institute	National Cancer Institute Implementation Science Centers in Cancer Control (ISC3)	USA / Maryland	https://cancercontrol.cancer.gov/is/initiatives/isc3	DI	Funding Research Seminars/Webinars Training/Courses Training Materials
National Cancer Institute	NCI Implementation Science Team	USA / Maryland	https://cancercontrol.cancer.gov/IS/	DI	Funding Research Seminars/Webinars Training/Courses Training Materials
National Institute on Aging IMbedded Pragmatic Alzheimer's disease and AD-Related Dementias Clinical Trials	National Institute on Aging IMbedded Pragmatic Alzheimer's disease and AD-Related Dementias Clinical Trials (NIA IMPACT) Collaboratory Implementation Core	USA	https://impactcollaboratory.org/about-us/cores-and-teams/implementation-core/	DI	Conferences/Workshops Database Funding Mentoring Research Training/Courses Training Materials Video Channel
National University of Singapore	Centre for Behavioural and Implementation Science Interventions	Singapore	https://medicine.nus.edu.sg/bisi/	DI	Conferences/Workshops Funding Research Seminars/Webinars Training/Courses
New York University	NYU School of Public Health - Global Center for Implementation Science	USA / New York	https://publichealth.nyu.edu/w/gcis	DI	Research Seminars/Webinars Training/Courses
Nigerian Implementation Science Alliance	Nigerian Implementation Science Alliance	Nigeria	https://nisaresearch.org	DI	Conferences/Workshops Mentoring Research Seminars/Webinars
Northwestern University	The Behavioral, Social, and Implementation Science (BSIS) Core	USA / Illinois	https://www.thirdcoastcfar.org/behavioral-social-core/	DI	Conferences/Workshops Consultation Data Analysis Funding Training/Courses
Oregon Health & Science University	BRIDGE-C2: Building Research in Implementation & Dissemination to Close Gaps & Achieve Equity in Cancer Control	USA / Oregon	https://bridgetoinnovation.org/	QI	Fellowships Framework/Tool Development Seminars/Webinars Training/Courses
SIRC	Society for Implementation Research Collaboration	USA / Arkansas	https://societyforimplementationresearchcollaboration.org	DI	Conferences/Workshops Mentorship Seminars/Webinars Training/Courses Training Materials
St. Michael's Hospital	Knowledge Translation Program	Canada / Ontario	https://knowledgetranslation.net/	KT	Conferences/Workshops Framework/Tool Development Seminars/Webinars Training/Courses
The Center for Implementation	The Center for Implementation	Canada / Ontario	https://thecenterforimplementation.com/	DI, KT	Conferences/Workshops Consultation Training/Courses Seminars/Webinars
The University of Adelaide	JBI Evidence Implementation Program	Australia	https://jbi.global/about-jbi	DI	Conferences/Workshops Consultation Database Framework/Tool Development Seminars/Webinars Training/Courses
The University of Illinois at Chicago	Community Engagement Core of the Center for Clinical Translational Science	USA / Illinois	https://ccts.uic.edu/about/	DI	Conferences/Workshops Consultation Framework/Tool Development Funding Mentoring Research Seminars/Webinars Training/Courses
The University of Iowa	Institute for Clinical and Translational Science Engagement, Integration, and Implementation Core	USA / Iowa	https://icts.uiowa.edu/	DI	Framework/Tool Development Funding Mentoring Research Seminars/Webinars Training/Courses
The University of North Carolina	North Carolina Translational and Clinical Sciences Institute (NC TraCS)	USA / North Carolina	https://tracs.unc.edu/	DI	Consultation Funding Framework/Tool Development Mentoring Research Seminars/Webinars Training/Courses
Tribal Evaluation Institute	Tribal Evaluation Institute	USA / Virginia	https://www.tribaleval.org/share-what-you-learned/	QI	Database Research Training Materials
UCSD	ACTRI Dissemination and Implementation Science Center (DISC)	USA / California	https://medschool.ucsd.edu/research/actri/centers/DIR/Pages/default.aspx	DI	Conferences/Workshops Consultation Funding Mentorships Research Seminars/Webinars Training/Courses
UCSF	IMPACT	USA / California	https://ctsi.ucsf.edu/	DI	Consultation Fellowships Funding Guideline Dev. Mentorship Research Seminars/Webinars Training/Courses
UCSF	Implementation Science and Health Systems Core	USA / California	https://prevention.ucsf.edu/about/ucsf-center-aids-prevention-studies-caps/caps-implementation-science-and-health-systems-core	DI	Conferences/Workshops Consultation Fellowships Research Training/Courses Training Materials
UCSF	Partnerships for Research in Implementation Science for Equity (PRISE) Center	USA / California	https://prise.ucsf.edu/	DI	Conferences/Workshops Funding Mentorship Research Seminars/Webinars Training/Courses
UCSF	UCSF Implementation Science Training Program	USA / California	https://epibiostat.ucsf.edu/implementation-science-program	DI	Mentorship Research Training/Courses
UIC	Center for Dissemination and Implementation Science (CDIS)	USA / Illinois	https://chicago.medicine.uic.edu/departments/academic-departments/medicine/research/cdis/	DI	Conferences/Workshops Consultation Research Training/Courses
UK Implementation Society (UK-IS)	UK Implementation Society (UK-IS)	United Kingdom	https://www.ukimplementation.org.uk/	DI	Conferences/Workshops Seminars/Webinars Training Materials
UNC	Dissemination and Implementation Methods Unit	USA / North Carolina	https://tracs.unc.edu/index.php/services/implementation-science	DI	Consultation Database Seminars/Webinars
UNC	National Implementation Research Network	USA / North Carolina	https://nirn.fpg.unc.edu/national-implementation-research-network	DI, QI	Conferences/Workshops Framework/Tool Development Guideline Dev. Research Seminars/Webinars Training/Courses
UNC	The Impact Center at FPG	USA / North Carolina	https://impact.fpg.unc.edu/	DI, QI	Data Analysis Framework/Tool Development Research Video Channel
University of Arkansas	UAMS Center for Implementation Research	USA / Arkansas	https://tri.uams.edu/resources-and-services/tri-services/the-center-for-implementation-research/	DI	Data Analysis Framework/Tool Development Funding Mentorship Research Training/Courses
University of Colorado	CCTSI Dissemination and Implementation Research Core	USA / Colorado	https://cctsi.cuanschutz.edu/	DI	Conferences/Workshops Consultation Fellowships Funding Mentoring Research Training/Courses
University of Colorado	Dissemination and Implementation Science Program, ACCORDS, Univ. Colorado SOM	USA / Colorado	https://medschool.cuanschutz.edu/accords/cores-and-programs/dissemination-implementation-science-program	DI	Consultation Fellowships Framework/Tool Development Mentorship Research Seminars/Webinars Training/Courses Training Materials
University of Oxford	Interdisciplinary Research in Health Sciences (IRIHS) research unit	United Kingdom	https://www.phc.ox.ac.uk/research/interdisciplinary-research-in-health-sciences	KT, QI	Consultation Framework/Tool Development Research Video Channel
University of Pennsylvania	Penn Implementation Science Center (PISCE@LDI)	USA / Pennsylvania	https://ldi.upenn.edu/about-us/partners/penn-ldi-affiliated-centers/penn-implementation-science-center/	DI	Conferences/Workshops Fellowships Seminars/Webinars Training/Courses
University of Pennsylvania	The Penn Collaborative for CBT and Implementation Science	USA / Pennsylvania	https://www.med.upenn.edu/penncollaborative/what-is-implementation.html	DI	Consultation Training/Courses Research
University of Pittsburgh	Clinical and Translational Science Institute (CTSI)	USA / Pennsylvania	https://ctsi.pitt.edu/	DI	Conferences/Workshops Fellowships Framework/ Tool Development Funding Mentoring Seminars/Webinars Training/Courses
University of Rochester	Equity-focused Dissemination and Implementation Function, University of Rochester CTSI	USA / New York	https://www.urmc.rochester.edu/clinical-translational-science-institute.aspx	DI	Consultation Framework/Tool Development Funding Mentoring Research Training/Courses
University of Texas Health Science Center at Houston	UT Health Institute for Implementation Science	USA / Texas	in development	QI	Consultation Funding Mentoring Research Seminars/Webinars
University of Texas Health Science Center at San Antonio	Institue for Integration of Medicine & Science Improvement Science Research Network	USA / Texas	https://isrn.net/https%3A//improvementscienceresearch.net/about	QI	Conferences/Workshops Data Analysis Research Training/Courses Training Materials Seminars/Webinars
University of Texas Health Science Center at San Antonio	Institute for Integration of Medicine & Science	USA / Texas	https://iims.uthscsa.edu/	DI	Conferences/Workshops Consultation Database Funding Mentorship Research Seminars/Webinars Training/Courses Training Materials
University of Utah	Clinical & Translational Science Institute (CTSI)	USA / Utah	https://ctsi.utah.edu/	DI	Conferences/Workshops Consultation Data Analysis Funding Guideline Dev. Mentoring Research Seminars/Webinars Training/Courses
University of Washington	Dissemination and Implementation Program, Institute of Translational Health Sciences	USA / Washington	https://www.iths.org/community/partners/d-i/	DI	Consultation Framework/Tool Development Research
University of Washington	Military Suicide Research Consoriuum Dissemination and Implementation Core (Core D)	USA / Washington	https://www.uwcspar.org/di.html	DI	Conferences/Workshops Consultation Framework/Tool Development Guideline Dev. Research Seminars/Webinars
University of Washington	The University of Washington Department of Global Health Implementation Science Program	USA / Washington	https://impsciuw.org/	DI	Mentorship Research Training/Courses
University of Washington	UW/Fred Hutch Center for AIDS Research Implementation Science Core	USA / Washington	http://depts.washington.edu/cfar/discover-cfar/cores/implementation-science	DI	Conferences/Workshops Consultation Mentorship Research Training/Courses
University of Wisconsin	Dissemination and Implementation Launchpad	USA / Wisconsin	https://ictr.wisc.edu/dissemination-implementation-launchpad/	DI	Consultation Framework/Tool Development Funding Research Training/Courses Training Materials
University of Zurich	Institute for Implementation Science in Health Care	Switzerland	https://www.ifis.uzh.ch/en.html	DI, QI	Consultation Framework/Tool Development Research Seminars/Webinars Training/Courses
USC	Center for Healthcare Delivery Science for Health Equity	USA / California	https://sc-ctsi.org/	DI	Conferences/Workshops Consultation Framework/Tool Development Funding Mentoring Seminars/Webinars Training/Courses Training Materials Video Channel
Vanderbilt University	Center for Clinical Quality and Implementation Research (CCQIR)	USA / Tennessee	https://www.vumc.org/implementation/center-clinical-quality-and-implementation-research	DI, QI	Fellowships Mentorship Research Training/Courses
Vanderbilt University	Vanderbilt Implementation and Quality Improvement Core	USA / Tennessee	https://www.vumc.org/implementation/VIQIcore	DI, QI	Consultation Data Analysis
Vanderbilt University	Vanderbilt Institute for Clinical and Translational Research - Dissemination Core	USA / Tennessee	https://victr.vumc.org/dissemination-of-research-results/	DI *CE*	Framework/Tool Development Research Training Materials
Veteran’s Affairs	Behavioral Health Quality Enhancement Research Initiative (QUERI) Program	USA	https://www.queri.research.va.gov/centers/Behavioral-Health.pdf	DI, QI	Consultation Mentorship Research Training/Courses
Veteran’s Affairs	Center for Care Delivery and Outcomes Research Implementation Core	USA	https://www.ccdor.research.va.gov/CCDORRESEARCH/CCDOR_Implementation.asp	DI	Conferences/Workshops Consultation Mentorship Research Framework/Tool Development Training/Courses
Veteran’s Affairs	EMPOWER QUERI	USA	https://www.queri.research.va.gov/centers/EMPOWER.pdf	DI, QI	Data Analysis Framework/Tool Development Research
Veteran’s Affairs	Expanding Expertise Through E-health Network Development (EXTEND) Telehealth QUERI Program	USA	https://www.queri.research.va.gov/centers/EXTEND.pdf	DI, QI	Data Analysis Framework/Tool Development Guideline Dev. Mentorship Research
Veteran’s Affairs	Function & Independence QUERI	USA	https://www.durham.hsrd.research.va.gov/Newsletters.asp	DI, QI	Data Analysis Research Training/Courses Training Materials
Veteran’s Affairs	High-RIsk VETerans (RIVET) QUERI Program	USA	https://www.queri.research.va.gov/centers/RIVET.pdf	DI, QI	Consultation Data Analysis Framework/Tool Development Mentorship Research Training/Courses
Veteran’s Affairs	Medication Safety (MedSafe) QUERI Program	USA	https://www.ci2i.research.va.gov/CI2IRESEARCH/docs/MedSafe_QUERI.pdf	DI, QI	Data Analysis Framework/Tool Development Research
Veteran’s Affairs	Measurement Science QUERI	USA	https://www.queri.research.va.gov/centers/MeasurementScience.pdf	DI, QI	Data Analysis Framework/Tool Development Research Training/Courses
Veteran’s Affairs	Quality Enhancement Research Initiative (QUERI) National Program	USA	https://www.queri.research.va.gov/	DI	Conferences/Workshops Consultation Data Analysis Framework/Tool Development Funding Mentorship Research Seminars/Webinars Training/Courses
Veteran’s Affairs	QUERI - Center for Evaluation and Implementation Resources (CEIR)	USA	https://www.queri.research.va.gov/ceir/default.cfm	DI, QI	Consultation Data Analysis Seminars/Webinars Training/Courses
Wake Forest School of Medicine	Department of Implementation Science	USA / North Carolina	https://school.wakehealth.edu/Departments/Implementation-Science	DI	Framework/Tool Development Research
Wake Forest School of Medicine	Wake Forest School of Medicine Implementation Science Affinity Group	USA / North Carolina	https://ctsi.wakehealth.edu/About-CTSI/CTSA-Programs/Implementation-Science-Affinity-Group	DI	Seminars/Webinars Training Materials
Washington University in St. Louis	Center for Dissemination and Implementation at the Institute for Public Health; Dissemination and Implementation Core in the Institute for Clinical and Translational Sciences at Washington University	USA / Missouri	https://publichealth.wustl.edu/centers/cdi/	DI	Conferences/Workshops Funding Research Training/Courses Training Materials
Washington University in St. Louis	HIV, Infectious Disease and Global Health Implementation Research Institute (HIGH-IRI)	USA / Missouri	https://sites.wustl.edu/highiri/	DI	Mentoring Research Training/Courses
Washington University in St. Louis	Institute of Clinical and Translational Sciences (ICTS)	USA / Missouri	https://icts.wustl.edu/	DI	Conferences/Workshops Consultation Funding Mentoring Research Seminars/Webinars Training/Courses Training Materials
Weill Cornell Medicine	Clinical & Translational Science Center (CTSC)	USA / New York	https://ctscweb.weill.cornell.edu/	DI	Consultation Framework/Tool Development Funding Mentoring Research Seminars/Webinars Training/Courses
Yale School of Public Health	Center for Methods in Implementation and Prevention Science (CMIPS)	USA / Connecticut	https://ysph.yale.edu/cmips/	DI	Mentorship Research Seminars/Webinars Training/Courses
Remaining Programs (*n*=79)					
Canadian Centre for Applied Research in Cancer Control	Canadian Centre for Applied Research in Cancer Control	Canada / British Columbia and Ontario	https://cc-arcc.ca/	KT	Conferences/Workshops Fellowships Mentorship Policy Research Seminars/Webinars Training Materials Work Placement
Canadian Institutes of Health Research	Strategy for Patient-Oriented Research (SPOR) Evidence Alliance	Canada	https://sporevidencealliance.ca	DI	Conferences/Workshops Mentorship Research Seminars/Webinars Training/Courses
Centre for Addiction and Mental Health	The Knowledge Exchange (KE) team at the Provincial System Support Program (PSSP)	Canada / Ontario	https://www.eenet.ca/knowledge-exchange-within-pssp	KT	Conferences/Workshops Seminars/Webinars Training/Courses
Centre for Effective Services	Centre for Effective Services (CES)	Ireland	https://www.effectiveservices.org/	DI, KT *Policy*	Conferences/Workshops Consultation Data Analysis Framework/Tool Development Research Training/Courses
Centre for Evidence and Implementation	Centre for Evidence and Implementation (CEI)	Australia	https://www.ceiglobal.org/	DI	Conferences/Workshops Data Analysis Framework/Tool Development Research
City University of London	Centre for Healthcare Innovation Research	United Kingdom	https://www.city.ac.uk/research/centres/healthcare-innovation?_ga=2.182974181.27301676.1635278687-1148846885.1635278687	QI *Policy*	Research Seminars/Webinars Training/Courses
City University of New York	Health Evaluation and Applied Research Development (HEARD)	USA / New York	https://www.heardproject.org/	DI	Data Analysis Framework/Tool Development Funding Research
CUNY	CUNY Institute for Implementation Science in Population Health	USA / New York	https://cunyisph.org/about/	DI	Mentorship Research Seminars/Webinars Training Materials
Duke University	Clinical & Translational Science Institute	USA / North Carolina	https://www.ctsi.duke.edu/	DI	Funding Internships Mentorship Research Seminars/Webinars Training/Courses
Duke University	INTERACT (Implementation Science Research Collaborative)	USA / North Carolina	https://populationhealth.duke.edu/research/implementation-science-research-collaborative-interact	DI	Mentorship Research
Einstein College of Medicine	Harold and Muriel Block Institute for Clinical and Translational Research (ICTR)	USA / New York	https://einsteinmed.edu/centers/ictr/	DI	Consultation Mentorship Research Seminars/Webinars Training/Courses
Global Alliance and Chronic Diseases (GACD) Wellcome Trust	Global Alliance and Chronic Diseases (GACD) Wellcome Trust	United Kingdom	https://www.gacd.org/community/capacity-development	DI	Conferences/Workshops Database Training/Courses
Global Implementation Society	Global Implementation Initiative	USA / North Carolina	https://globalimplementation.org/	DI	Conferences/Workshops Seminars/Webinars Training Materials
Harvard School of Public Health	The Implementation Science Center for Cancer Control Equity - Research Program	USA / Massachusetts	https://reporter.nih.gov/project-details/9869341	DI	Data Analysis Framework/Tool Development Research
Health Foundation Improvement Science Development Group	Health Foundation Improvement Science Development Group	United Kingdom	http://health.org.uk/	QI	Conferences/Workshops Data Analysis Database Fellowships Funding Research Seminars/Webinars Training/Courses
Health Systems Global	Health Systems Global	Canada / Ontario	https://healthsystemsglobal.org/	KT	Conferences/Workshops Database Mentorship Seminars/Webinars Training/Courses
Healthcare Quality Improvement Partnership (HQIP)	Healthcare Quality Improvement Partnership (HQIP)	United Kingdom	https://www.hqip.org.uk/about-us/#.YKQ8fZNKhfU	KT, QI	Consultation Database Research Training/Courses
Improvement Science London	Improvement Science London	United Kingdom	https://www.ucl.ac.uk/epidemiology-health-care/research/primary-care-and-population-health/research/isl	QI	Fellowships Research Seminars/Webinars
Institute of Health Economics Alberta, CA	Knowledge Transfer & Dissemination	Canada / Alberta	https://www.ihe.ca/research-programs/knowledge-transfer-dissemination	KT, DI	Conferences/Workshops Training/Courses
Kansas City Quality & Value Innovation Consortium	Kansas City Quality and Value Innovation Consortium	USA / Missouri	https://www.kcqvic.org/	DI *CE*	Research Training/Courses
King’s College London	King’s Improvement Science	United Kingdom	https://kingsimprovementscience.org/	DI, QI	Framework/Tool Development Research
Kingston University London	Implementation and Improvement Research Group	United Kingdom	https://www.healthcare.ac.uk/research/implementation-and-improvement-research-group/	DI	Mentorships Research Seminars/Webinars
Knowledge Translation Alberta	Knowledge Translation Alberta	Canada / Alberta	https://www.ktalberta.ca/	KT	Consultation Seminars/Webinars Training/Courses
London School of Economics	London School of Economics - Knowledge Exchange and Impact	United Kingdom	https://www.ihe.ca/research-programs/knowledge-transfer-dissemination	KT, DI	Conferences/Workshops Consultation Research Training/Courses Training Materials
Mayo Clinic	Mayo Clinic Center for Clinical and Translational Science (CCaTS) - D&I Core	USA / Minnesota	https://www.mayo.edu/research/centers-programs/center-clinical-translational-science/resources/consultative-resources/dissemination-and-implementation-science	DI	Consultation Mentorship Research Training/Courses Training Materials
Medical University of South Carolina	South Carolina Clinical & Translational Research Institute - The Dissemination & Implementation Science Collaborative	USA / South Carolina	https://research.musc.edu/resources/sctr/programs/disc	DI	Consultation Conferences/Workshops
Medstar Health	Medstar Health	USA / Maryland	https://www.medstarhealth.org/innovation-and-research/institute-for-quality-and-safety	QI	Conferences/Workshops Research Training/Courses
Michigan State University	Dissemination and Implementation Core	USA / Michigan	http://www.flintcenter.org/our-cores/dissemination-and-implementation-science-core/	DI *CE*	Data Analysis Research
Monash	Monash Centre for Health Research and Implementation (MCHRI)	Australia / Victoria	https://www.monash.edu/medicine/sphpm/mchri/home	DI, KT	Framework/Tool Development Research Training/Courses
National Cancer Center	Research Application for Dissemination and Implementation Science in Health (RADISH)	Japan	https://www.radish-japan.org/en/about/index.htm	DI	Conferences/Workshops Research Training/Courses
National Centre of Implementation Science	National Centre of Implementation Science	Australia	https://ncois.org.au/home-page/about-us/	DI	Conferences/Workshops Research Seminars/Webinars Training/Courses Training Materials
New York University	NYU Langone Health - Clinical & Translational Science Institute (CTSI)	USA / New York	https://med.nyu.edu/departments-institutes/clinical-translational-science/	DI	Conferences/Workshops Consultation Funding Mentorship Research Seminars/Webinars Training/Courses
New York University	NYU Langone Health - The IDEAS Center	USA / New York	http://www.ideas4kidsmentalhealth.org/	DI *Policy*	Fellowship Framework/Tool Development Research Seminars/Webinars Training/Courses
Northwestern University Feinberg School of Medicine	Ce-PIM: Center for Prevention Implementation Methodology	USA / Illinois	https://www.cepim.northwestern.edu	DI	Conferences/Workshops Consultation Research Training/Courses
Northwestern University Feinberg School of Medicine	Northwestern University Clinical and Translational Sciences Institute - Dissemination and Implementation Program	USA / Illinois	https://www.nucats.northwestern.edu/about/centers-and-programs/dissemination-implementation-program.html	DI	Consultation Framework/Tool Development Funding Seminars/Webinars Training/Courses
Rockefeller University	Center for Clinical and Translational Science	USA / New York	https://www2.rockefeller.edu/ccts/	DI	Mentoring Research Seminars/Webinars Training/Courses
Royal College of Psychiatrists	College Center for Quality Improvement (CCQI), Royal College of Psychiatrists	United Kingdom	https://www.rcpsych.ac.uk/improving-care/ccqi	QI	Conferences/Workshops Consultation Research Seminars/Webinars Training/Courses
Rutgers (New Brunswick, Newark and Camden), Princeton and New Jersey Institute of Technology (NJIT)	New Jersey Alliance for Clinical Translational Science	USA / New Jersey	https://njacts.rbhs.rutgers.edu/	DI	Conferences/Workshops Consultation Data Analysis Funding Research Seminars/Webinars Training/Courses
Stanford University	Clinical and Translational Research Unit (CTRU)	USA / California	https://med.stanford.edu/ctru.html	DI	Research Training Materials
Stellenbosch University	Center for Evidence Based Health Care - Knowledge Translation	South Africa	https://www.cebhc.co.za/knowledge-translation-what-we-do/	DI, KT	Conferences/Workshops Framework/Tool Development Research Training Materials
Swiss Implementation Science Network (IMPACT)	Swiss Implementation Science Network (IMPACT)	Switzerland	https://impact-dph.unibas.ch/	DI	Conferences/Workshops Training/Courses Seminars/Webinars
The Hospital for Sick Children (SickKids)	The Hospital for Sick Children (SickKids) - Knowledge Translation Program	Canada / Ontario	https://www.sickkids.ca/en/learning/our-programs/knowledge-translation-program/	DI, KT	Conferences/Workshops Framework/Tool Development Training/Courses Training Materials
The University of Chicago - Center for Healthcare Delivery Science and Innovation (HDSI)	The University of Chicago - Center for Healthcare Delivery Science and Innovation (HDSI)	USA / Illinois	https://hdsi.uchicago.edu/about-hdsi/	DI, QI	Conferences/Workshops Consultation Fellowships Funding Mentoring Research Training/Courses
The University of Florida	Clinical and Translational Science Institute (CTSI)	USA / Florida	https://www.ctsi.ufl.edu/	DI	Conferences/Workshops Consultation Data Analysis Database Funding Mentoring Research Seminars/Webinars Training/Courses
The University of Texas Houston	The University of Texas Health Science Center at Houston (UT Health) Center for Clinical and Translational Science (CCTS)	USA / Texas	https://www.uth.edu/ccts/	DI	Consultation Funding Framework/Tool Development Mentoring Research Seminars/Webinars Training/Courses
TMF Health Quality Institute	Quality Innovation Network-Quality Improvement Organization (QIN-QIO)	USA / Texas	https://tmf.org/Our-Work/Quality-Improvement	QI	Consultation Framework/Tool Development Research Training/Courses
University of Massachusetts	Implementation Science & Practice Advances Research Center (iSPARC)	USA / Massachusetts	https://www.umassmed.edu/sparc/	DI, KT *CE*	Conference/Workshops Consultation Fellowships Research Seminars/Webinars
UC Davis	UC Davis Clinical and Translational Science Center (CTSC) - Translational Resources and Pilot Program	USA / California	https://health.ucdavis.edu/ctsc/area/translationalresearch/index.html	DI	Conference/Workshops Data Analysis Funding Mentoring Research Training/Courses
UCLA	Clinical and Translational Science Institute (CTSI) - Community Engagement and Research Program	USA / California	https://www.ctsi.ucla.edu/patients-community/pages/dissemination_implementation_improvement	DI	Conferences/Workshops Consultation Data Analysis Database Fellowships Funding Research Seminars/Webinars Training/Courses
UCSF	Research in Implementation Science for Equity (RISE)	USA / California	https://cvp.ucsf.edu/programs/capacity-building-training/research-implementation-science-equity-rise	DI	Conferences/Workshops Funding Mentorship Research Training/Courses
UNC	Center for Health Promotion and Disease Prevention (HPDP) - Translation and Dissemination Service	USA / North Carolina	https://hpdp.unc.edu/research-services/communication-dissemination/	DI	Research Training Materials
UNC	Consortium for Implementation Science - RTI International and UNC Gillings School of Global Public Health	USA / North Carolina	https://consortiumforis.org/	DI	Conferences/Workshops Research
UNC	Frank Porter Graham (FPG) - Child Development Institute - National Implementation Research Network (NIRN) - Active Implementation Hub (AI Hub)	USA / North Carolina	https://nirn.fpg.unc.edu/ai-hub	DI	Seminars/Webinars Training/Courses
UNC	Implementation Science Exchange	USA / North Carolina	https://impsci.tracs.unc.edu/	DI	Database Framework/Tool Development Training/Courses Training Materials
UNC	Institute for Healthcare Quality Improvement (IHQI)	USA / North Carolina	https://www.med.unc.edu/ihqi	QI	Conferences/Workshops Fellowships Mentorship Research Training/Courses Training Materials
University College London	Centre for Behavior Change (CBC)	United Kingdom	https://www.ucl.ac.uk/behaviour-change/	DI	Conferences/Workshops Consultation Framework/Tool Development Guideling Dev. Research Seminars/Webinars Training/Courses Training Materials
University of Cambridge	The Healthcare Improvement Studies Institute (THIS) at the U of Cambridge	United Kingdom	https://www.thisinstitute.cam.ac.uk/	DI	Conferences/Workshops Fellowships Funding Mentorship Research Seminars/Webinars
University of Chicago	Chapin Hall’s Implementation Collaborative	USA / Illinois	https://www.chapinhall.org/project/implementation-collaborative/	QI *Policy*	Data Analysis Framework/Tool Development Research Seminars/Webinars Training/Courses
University of Chicago	The University of Chicago: Center for Healthcare Delivery Science and Innovation (HDSI)	USA / Illinois	https://hdsi.uchicago.edu/	QI	Conferences/Workshops Consultation Data Analysis Fellowships Funding Mentorship Research Training/Courses
University of Cincinnati	Center for Improvement Science (CIS)	USA / Ohio	https://www.cctst.org/programs/cis	QI	Conferences/Workshops Consultation Data Analysis Research Training/Courses Training Materials
University of East Anglia	Behavioural and Implementation Science group	United Kingdom	https://www.uea.ac.uk/about/school-of-health-sciences/research/behavioural-and-implementation-science	DI	Consultation Funding Internships Research Seminars/Webinars Training/Courses
University of Edinburgh	The University of Edinburgh: Edinburgh Research Office Knowledge Exchange and Impact Team	Scotland	https://www.ed.ac.uk/research-office/about/what-we-do/knowledge-exchange-and-impact-team	KT	Conferences/Workshops Consultation Funding Research Training/Courses
University of Leicester	Social Science Applied to Healthcare Improvement Research (SAPPHIRE)	United Kingdom	https://le.ac.uk/sapphire	KT, QI	Mentorship Research Training/Courses
University of Melbourne	Melbourne School of Population and Global Health (MSPGH)	Australia	https://mspgh.unimelb.edu.au/centres-institutes/nossal-institute-for-global-health/implementation-science#home	DI	Research Training/Courses Training Materials
University of Montreal	Institute of Gender and Health	Canada / Québec	https://cihr-irsc.gc.ca/e/40948.html	KT	Conferences/Workshops Funding Seminars/Webinars Training/Courses Training Materials
University of Oslo	Knowledge in translation (KNOWIT)	Norway	https://www.med.uio.no/helsam/english/research/groups/knowit/	KT	Framework/Tool Development Guideline Dev. Research
University of Southampton	Wessex Centre for Implementation Science	United Kingdom	https://www.southampton.ac.uk/wessexcis/cis-projects/our-work.page	DI	Conferences/Workshops Data Analysis Framework/Tool Development Research Training Materials
University of Toronto	Ontario Pharmacy Evidence Network	Canada / Ontario	https://open-pharmacy-research.ca/research/quality-improvement-in-pharmacy-practice	DI, QI	Framework/Tool Development Research
Veteran’s Affairs	QUERI - Age Friendly Health System	USA	https://www.queri.research.va.gov/centers/Age-Friendly.pdf	DI, QI	Mentoring Research Training/Courses
Veteran’s Affairs	QUERI - Antimicrobial Stewardship (CARRIAGE)	USA	https://www.queri.research.va.gov/centers/CARRIAGE.pdf	DI, QI	Data Analysis Research Training/Courses
Veteran’s Affairs	QUERI - Bridging the Care Continuum (Bridge)	USA	https://www.queri.research.va.gov/centers/Bridge.pdf	DI, QI	Data Analysis Mentoring Research Training/Courses
Veteran’s Affairs	QUERI - Dynamic Diffusion Network (DDN)	USA	https://www.durham.hsrd.research.va.gov/Dynamic_Diffusion_Network_DDN_QUERI_Program.asp	DI, QI	Fellowships Data Analysis Mentorship Research Training/Courses
Veteran’s Affairs	QUERI - Quadruple Aim	USA	https://www.queri.research.va.gov/centers/QuadrupleAim.pdf	DI, QI	Data Analysis Research
Veteran’s Affairs	QUERI - Virtual Care	USA	https://www.queri.research.va.gov/centers/VirtualCare.pdf	DI, QI	Framework/Tool Development Research
Wake Forest School of Medicine	Clinical and Translational Science Institute (CTSI)	USA / North Carolina	https://ctsi.wakehealth.edu/	DI	Consultation Fellowships Funding Mentoring Research Seminars/Webinars Training/Courses
Western University	Western Research Knowledge Exchange & Impact	Canada / Ontario	https://www.uwo.ca/research/services/kex/index.html	KT	Consultation Framework/Tool Development Research Training/Courses Training Materials
Worcestershire Acute Hospitals	Worcestershire Acute Hospitals Quality Improvement Team	England	https://www.worcsacute.nhs.uk/quality-improvement/quality-improvement-team	QI	Mentorship Research Seminars/Webinars Training/Courses
World Health Organization	Alliance for Health Policy and Systems Research: Implementation Research and Delivery Science (IRDS) Collaboration	Switzerland	https://www.who.int/alliance-hpsr/projects/irds/en/	DI *Policy*	Conferences/Workshops Fellowships Research Seminars/Webinars Training Materials

**Table 3:** Dissemination and implementation science capacity building activities

Activity	Definition
Conferences/Workshops	Formal gatherings or professional working meetings about specific DIS topics
Consultation	Expert review and feedback on DIS projects or questions
Data analysis	Support in organizing, analyzing, and interpreting DIS data
Database	Searchable DIS-related data repository
Fellowships	Additional education and support for graduate students
Framework/Tool Development	Creation of models, frameworks, or tools to support DIS
Funding	Financial support for DIS research or practice
Guideline Development	Development of principles, objectives, or regulations to guide clinical care or research to improve quality
Internships	Work experience opportunities for students or trainees
Mentorships	Providing guidance in professional career advancement
Research	Systematic investigation to understand the environmental, genetic, and social health determinants that influence population health
Seminars/Webinars	Online or in-person lectures and speaking events
Training/Courses	Training, coursework or degree programs
Training Materials	Materials such as worksheets, diagrams, slide decks, informational guides or manuals
Video Channel	Training videos offered online (e.g., via YouTube)
Work Placement	Employment assistance or job placement services

### Phase 5—DIS capacity building survey distribution and findings

To gather additional information about each CBP, we emailed a Capacity Building Survey that requested detailed information about various aspects of the program’s capacity building efforts to the primary contact identified for each program, with up to three reminder emails. The survey was structured following the domains of the Washington University Network of Dissemination and Implementation Research model [1]. Model domains include inputs (e.g., funding model, human resources), activities (e.g., training, mentorship), outputs (e.g., grant outcomes, academic outcomes), and long-term public health outcomes guided by the Translational Science Benefits Model [20]. The following elements were also included in the survey: year when the CBP was established; the CBP’s primary contact; member characteristics, community partners, financial resources, types of activities, measurement, evaluation, and D&I competencies or frameworks for program evaluation (see Additional file 2 for the full survey). Outputs also included whether CBPs specialized in a DIS product or resource, with respondents able to elaborate in a text response. Results of the survey were analyzed by summarizing responses, displaying frequencies, and synthesizing themes. The number of respondents varied across survey items, so denominators reflect who responded to the respective item.

### Quality assurance assessment

We developed a rigorous process of multiple checks in which programs included and excluded were reviewed several times by independent team members. The process involved a first review by trained research assistants who established an agreement rate across other reviewers of at least 90% on a subsample of programs before initiating independent review. A sample of 100 search results were used to establish inter-rater agreement with >90% agreement serving as the threshold for concordance among reviewers. Second, the study coordinator reviewed all included sites for accuracy of inclusion and exclusion and also screened for duplicates. Next, there was a tertiary review by two senior team members in which each member independently reviewed 50% and then crosschecked each other’s decisions with a select number flagged for discussion. The refined list was circulated to a purposive sample of DIS experts for review and determination of missing sites. Lastly, programs were checked for accuracy and validity through a website check and abstraction of key data from websites.

## Results

### DIS capacity building program search results (Phases 1–3)

**Fig. 2:**
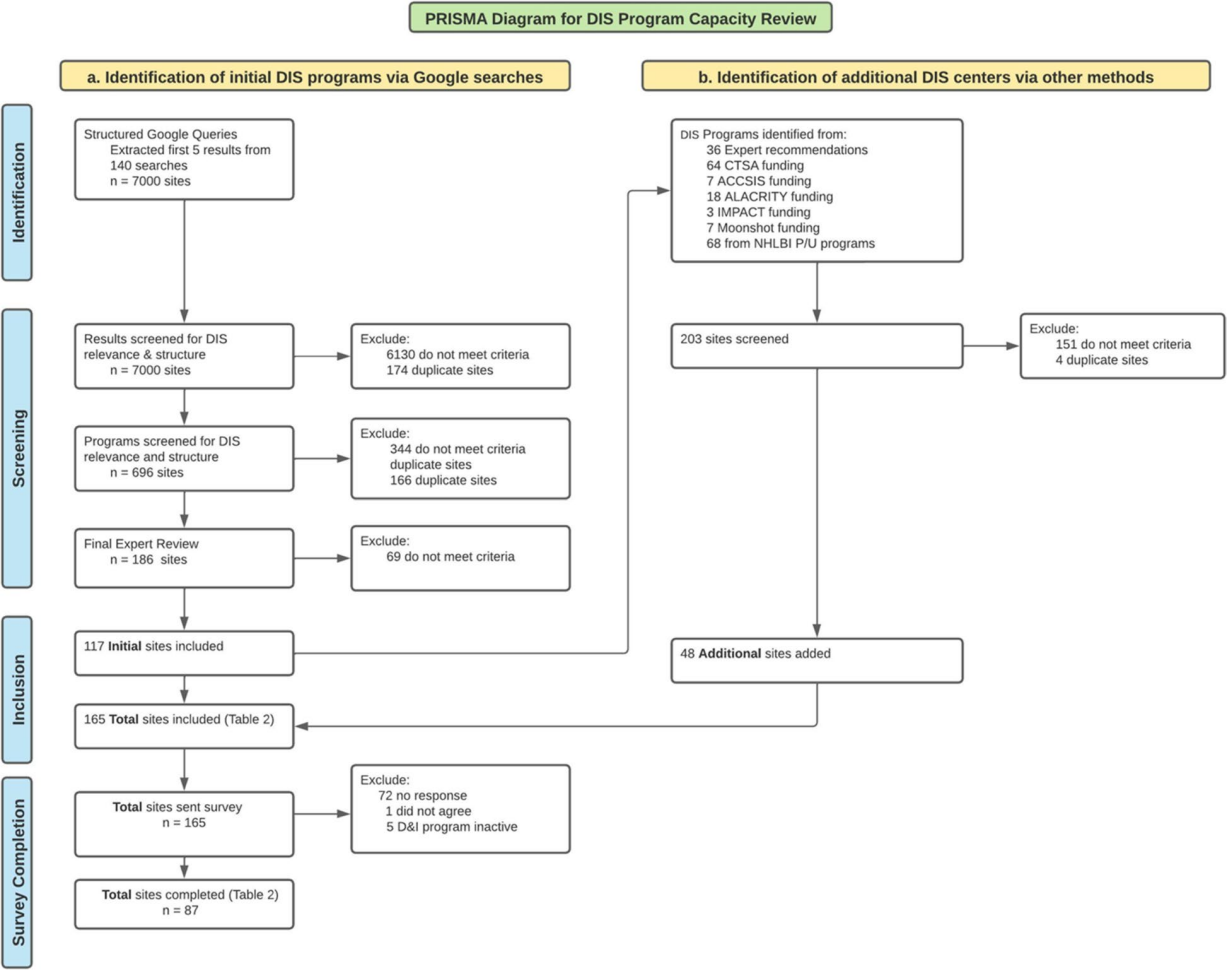
PRISMA diagram for DIS program systematic review

The first 50 search results were extracted from each unique search resulting in 140 searches and 7000 search results. After removing duplicates (*n*= 174) and those not meeting the eligibility criteria (*n*= 6130), 696 CBPs were retained (see Fig. 2). The secondary review for inclusion and duplicates resulted narrowed the sample to 186 CBPs. The tertiary review by faculty researcher team members resulted in an additional exclusion of 69 CBPs. The refined list of 117 programs was circulated to eight DIS experts who nominated 36 additional programs. Individual program searches of funding mechanisms yielded 162 additional programs. In total, through expert nomination and funding searches, an additional 203 programs were identified. Of these 203 CBPs, 151 did not meet criteria and 4 were duplicate sites. This phase resulted in 48 additional DIS CBPs for a total of 165 CBPs.

### DIS program characteristics (Phase 4)

One hundred sixty-five DIS CBPs are included in the final list displayed in Table 2. One hundred twelve (68%) are in the USA and 53 (32%) are internationally based. Program characteristics were abstracted from program websites and from responses of those CBPs who completed the follow-up survey (Table 2). One hundred thirty-one CBPs had a concentration in *Dissemination and Implementation Science (DIS)* (79%), 46 had a concentration in *Quality Improvement* (28%), 27 in *Knowledge Translation* (16%), 5 in *Policy* (3%), and 4 in *Community Engagement* (2%). Table 2 is organized alphabetically by host institution, with CBPs that responded to the survey highlighted at the top. Activities identified from website review included the following: Conferences/Workshops, Consultation, Data Analysis, Database, Fellowships, Framework/Tool Development, Funding, Guideline Development, Internships, Mentorship, Research, Seminars/Webinars, Training/Courses, Training Materials, Video Channel, and Work Placement.

### Capacity building survey results (Phase 5)

Ninety-two (56%) of the 165 CBPs invited responded to the survey invitation and 5 of 92 indicated that they were no longer active. Thus, 87 (53%) CBPs completed the survey. Of these, 62 (80%) were based in the USA and 56 (71%) were affiliated with an academic institution (see Table 4). Survey results indicated that 37 (43%) CBPs do not serve a specific population. Of those that specify a population of focus, 31 (36%) reported serving Adults, 29 (33%) reported Clinical, 27 (31%) Urban, 27 (31%) Women, 27 (31%) Older adults, and 23 (26%) General community. See Table 4 for a full summary of the Capacity Building Survey responses, and Additional file 1 displays qualitative responses to the question about program-specific DIS products or resources.

**Table 4:** DIS capacity building program (CBP) survey results

CBP characteristics	***n*** (%)
**Based in United States (*n*=78)**	62 (80%)
International	16 (20%)
**University-affiliated (*n*=79)**	56 (71%)
Total respondents^a^	87 (100%)
**Inputs**	
**Organizational structure**	
**# Faculty (*n*** **= 87)**	
0–5	34 (39%)
6–20	24 (28%)
21+	5 (6%)
**# Staff (*n*** **= 87)**	
0–5	42 (48%)
6–20	21 (24%)
21+	3 (3%)
**Membership structure (*n*=62)**	
0–50	22 (36%)
51–100	11 (18%)
101–150	3 (5%)
151–200	2 (3%)
201+	21 (34%)
Not sure	3 (5%)
**D&I funding model (*n*=73)**	
Short term (i.e., start-up funds)	9 (12%)
Long term (i.e., ongoing)	33 (45%)
Both	21 (29%)
Other	9 (12%)
**Sources of financial support (*n*=87)**	
Research / program grants	45 (52%)
Internal institutional funds	35 (40%)
CTSA	25 (29%)
Internal department funds	20 (23%)
Non-profits	17 (20%)
Government	9 (10%)
Education/course fees	4 (5%)
Membership fees	3 (4%)
Fees for services	3 (4%)
Other	4 (5%)
**Activities**	
**D&I activities (*n*=87)**	
Training and Education	69 (79%)
Mentorship	58 (67%)
Resources and Tools	57 (66%)
Consultation	58 (67%)
Professional Networking	54 (62%)
Technical Assistance	46 (52%)
Grant Development Support	45 (52%)
Internship for Students/Trainees	24 (28%)
Other	5 (6%)
**Types of training (*n*=69)**	
Webinars/Seminars	63 (91%)
Workshops	48 (70%)
Coursework	43 (62%)
Invited Guest Speakers	41 (59%)
Masters Programs (D&I or public health)	10 (14%)
Integrated training in medical programs	10 (14%)
Doctoral/PhD (D&I or public health)	9 (13%)
Other	8 (12%)
**Fees for D&I consultation services (*n*=58)**	
Ad hoc fees	5 (9%)
Situational	23 (40%)
No fees	28 (49%)
**D&I professional networking opportunities offered (*n*=54)**	
Virtual/In-person networking events	35 (65%)
Professional networking conferences	25 (46%)
Informal gatherings/meet-ups	22 (41%)
Journal club style meetings	19 (35%)
Other	13 (24%)
**Types of D&I grant development offered (*n*=45)**	
In-person/web-based trainings	28 (62%)
Works in Progress meetings	23 (51%)
Working groups	20 (44%)
Recorded video instructions	12 (27%)
Other	15 (33%)
**Types of D&I technical assistance offered (*n*=46)**	
In-person or virtual training	35 (76%)
Recorded video tutorials/trainings	23 (50%)
Working groups	19 (41%)
Journal club	13 (28%)
Other	8 (17%)
**Types of D&I resources and tools offered (*n*=57)**	
Educational materials	49 (86%)
Training videos	32 (56%)
Interactive web-based resources	30 (53%)
Other	15 (26%)
**Outputs**	
**D&I product (*n*=75)**^**b**^	
Yes	47 (63%)
No	28 (37%)
**Short-term outcomes**	
**D&I competencies (*n*=73)**	
Yes	27 (37%)
No	19 (26%)
Sometimes	17 (23%)
Not sure	10 (14%)
**Special populations of focus (*n*=87)**	
No special population	37 (43%)
Adults	31 (36%)
Clinical	29 (33%)
Women	27 (31%)
Urban	27 (31%)
Older adults	27 (31%)
General community	23 (26%)
Rural	23 (26%)
LGBTQ+	20 (23%)
Pediatrics	17 (20%)
Underserved/marginalized community	17 (20%)
Houseless/Homeless	14 (17%)
Indigenous communities	12 (14%)
Specific ethnic/racial group	9 (10%)
Other	11 (13%)
**Program evaluation domains (*n*=87)**	
Productivity	48 (55%)
Member engagement	32 (37%)
Member satisfaction	31 (36%)
Training effectiveness	30 (35%)
D&I Knowledge	20 (23%)
D&I skills	13 (15%)
Other	10 (12%)
**Productivity measurements (*n*=87)**	
# of Publications	56 (64%)
# of Grants	51 (59%)
# of Mentees	41 (47%)
# of Proposals submitted	33 (38%)
# of Active Members	31 (36%)
# of New collaborations	26 (30%)
Member Satisfaction	25 (29%)
Social media analytics	23 (27%)
# of Meetings hosted	22 (25%)
# of Individuals receiving communications	18 (21%)
# of Conferences hosted	17 (20%)
# Consultations	8 (9%)
Uptake of findings	8 (9%)
# Products	4 (5%)
Other	5 (6%)
**Frequency of productivity measurements (*n*=72)**	
Once a month	5 (7%)
Every 3 months	8 (11%)
Every 6 months	16 (22%)
Once a year	35 (49%)
Other	8 (11%)
**Long-term outcomes**	
**Use of Translational Science Benefits Indicators (TSBI) for evaluation (*n*=74)**	
Yes	20 (27%)
No	37 (50%)
Not sure	17 (23%)
**TSBI Categories (*n*=20)**	
Clinical & Medical	9 (45%)
Community & Public Health	13 (65%)
Economic	11 (55%)
Policy & Legislative	11 (55%)

^a^Responded to the first survey question with “I agree” to complete the survey and share responses for publication

^b^Responses included in Additional file 1

### DIS program inputs (Phase 5)

Most CBPs, 34 (39%), reported having 0 to 5 faculty with formal positions. Similarly, most CBPs, 42 (48%), reported having 0–5 staff with formal positions. A few CBPs reported having high numbers of faculty or staff; 5 (6%) CBPs had 21 or more faculty positions; 3 (3%) of CBPs had 50 or more; and 1 (1%) CBP had more than 50 staff positions. Regarding membership, 21 (34%) reported that their program had more than 200 members, whereas 22 (36%) reported 0–50 members. In terms of CBP funding, 33 (45%) described their funding sources as long term/ongoing, while 21 CBPs (29%) had both short term (project-specific or start-up funds) and long term. Specific sources of funding were research/program grants, 45 (52%), CTSA funding, 25 (29%), and internal institutional funds, 35 (40%).

### DIS program activities (Phase 5)

Sixty-nine (79%) provided Training and Education, 58 (67%) provided Mentorship, 57 (66%) offered Resources and Tools, and 58 (67%) provided Consultation. CBPs provided many types of DIS Training, including Webinars/Seminars 63 (91%), Coursework 43 (62%), and Invited Guest Speakers 41 (59%). Twenty-eight (49%) CBPs do not charge fees for DIS Consultations and 23 (40%) reported that consultation fees varied depending on the situation. Virtual/in-person Networking Events were the most common (35 (65%)) type of DIS Professional Networking opportunity offered. Of the CBPs that offered DIS grant development support, 28 (62%) held Virtual/in-person Training. Most DIS Technical Assistance was offered in the form of Virtual/in-person (35 (76%)) or recorded video tutorials/training (23 (50%)). Forty-nine (86%) CBPs offered educational materials as the most common type of DIS Resource and Tool.

### DIS program outputs and outcomes (Phase 5)

Outputs included whether CBPs specialized in a DIS product or resource. Of the 47 (63%) CBPs that affirmed the development of a DIS product, responses ranged across courses (10 (21%)) like the *Healthcare Delivery Science Course*, DIS models (14 (30%)) like the *Iowa Model for Evidence-based practice*, and DIS frameworks (10 (21%)) such as *StrategEase.* In terms of short-term outcomes and evaluation, 44 (60%) CBPs reported using DIS competencies to guide activities. Forty-eight CBPs (55%) evaluated their impact using Productivity measures, while Member Engagement (37%), Member Satisfaction (36%), and Training Effectiveness (35%) were also popular evaluation metrics. Thirty-five CBP (49%) respondents measure productivity once a year and 16 (22%) measure every 6 months.

Long-term outcomes include using Translational Science Benefits (TSB) Indicators or categories for evaluation [20, 21]. Twenty-seven percent of respondents (*n*=20) reported that they use the TSB indicators for evaluation. Of these, 13 (65%) reported using Community & Public Health (e.g., health care delivery, accessibility, life expectancy), 11 (55%) Economic (e.g., cost effectiveness, cost savings, societal cost of illness), 11 (55%) Policy and Legislative (e.g., committee participation, policies, expert testimony), and 9 (45%) Clinical and Medical indicators (e.g., drugs or diagnostic guidelines).

## Discussion

This systematic review identified 165 national and international DIS CBPs with most having more than two relevant DIS capacity building activities. As a result of this work, an interactive, searchable, online resource with an inventory of the programs will be made available for the DIS community to facilitate on-going capacity building, multidisciplinary engagement, and minimization of duplication of efforts across DIS CBPs. We also describe the key features of DIS CBPs as reported through the Capacity Building Survey. This study found that CBPs in the review described diverse funding models from several sources. Most CBPs have funding from research and program grants, as well as internal institutional grants and CTSA funding. The majority highlighted a robust infrastructure with faculty having formal program supervisory or operational roles. Three longstanding CBPs, Quality Enhancement Research Initiative (QUERI) National Program, National Cancer Institute Implementation Science Centers in Cancer Control (ISC3), Institute for Healthcare Improvement reported having more than 100 faculty with roles to support their DIS CBPs underscoring the immense human resources needed to build, deliver, and sustain large-scale DIS CBPs as compared to most respondents who reported fewer than 5 faculty. These CBPs are certainly outliers but nonetheless highlight that infrastructural funding is indispensable to the operations and delivery of CBPs.

There is overlap across capacity building activities and initiatives. Nearly all offer educational webinars or seminars in DIS consistent with exponential increases globally in virtual educational offerings, particularly since the COVID-19 pandemic. One conservative estimate suggests between 2020 and 2021 the number of webinars (not DIS-specific) grew by 162% and attendees increased 251% compared with before the pandemic [22]. Given that online seminars are often open to international and public audiences, there is a need to coordinate and streamline virtual programming so that there are fewer, more focused and targeted skill-building sessions to conserve resources. Anecdotally from our own center at UC San Diego, members highlight feeling overwhelmed and inundated with online seminars and unable to identify which might be most important, relevant, or applicable to their work.

Mentorship and consultation were frequently used capacity building strategies underscoring the potential critical importance of individualized and tailored guidance when applying DIS approaches. Despite most CBPs offering consultation, these approaches may be more difficult to grow at scale and unable to accommodate growing interest among trainees, staff, faculty, and D&I practitioners. Compared with the other capacity building strategies reported by 70–94% of CBPs, relatively fewer reported offering technical assistance (65%) as one of their CBP activities. Understanding why technical assistance is offered by fewer CBPs might be an important question for future study considering that technical assistance often includes providing hands-on support to community partners and other stakeholders, an area critical for operationalizing DIS [23].

Few CBPs reported offering Doctoral or PhD programs with a focus on DIS. As DIS evolves and expands, there is arguably a greater need for formalized coursework and programmatic offerings within higher-education degree programs as compared with offering traditional single seminar, workshops, and virtual offerings. DIS users may also want more formal training activities (e.g., those that offer a certification of completion) so they can be considered credentialed implementation scientists and include this in their professional curriculum vitae.

Furthermore, few programs endorsed offering DIS training within clinical professional degree programs, illustrating the gap in the field of DIS approaches in clinical practice. Clinical researchers and practitioners tend to have interest in DIS and use DIS approaches in their research, and would likely benefit from the integration of DIS coursework into their degree programs [24, 25]. There are numerous resources available online for training and education in DIS, but many of these existing opportunities available may be best suited for early career investigators or trainees who are still forming their careers and research interests. There is a need to support mid to later stage investigators who may not have time to independently study DIS and integrate DIS into their research, requiring more individualized and tailored guidance. These findings align with the Davis & D’Lima [2] review of training institutes; there appear to be fewer opportunities for later-stage investigators, practitioners, policy makers, and community partners despite a growing number of degree or certificate programs [26] such as University of Washington’s PhD in global health metrics and implementation science [27] or University of California San Francisco Implementation Science Certificate program [28].

This review also revealed a dearth of programs across LMICs. Only one CBP, the Nigerian Implementation Science Alliance, was identified in sub-Saharan Africa. We did not locate programs in South America, Central America, or Eastern Europe. It is also noteworthy from our review that DIS CBPs necessitate immense resources, time, and personnel to plan, market, and deliver training, networking, consultation, and other DIS opportunities for researchers and practitioners. Institutions based in LMICs may have fewer disposable resources to devote to DIS capacity building. Moreover, with the range and diversity of DIS offerings, it is increasingly difficult to know whether CBPs are effective in producing measurable impact and ultimately improving population health. There is a need for development of shared outcomes and success metrics across DIS CBPs which will not only advance programs’ ability to advocate for funding but will facilitate broad evidence synthesis to identify programs with high degree of output and impact. Davis & D’Lima also find that standardized metrics are needed in the reporting of key elements of DIS training content and structure as well as the evaluation of the CBPs [2].

Strengths of this systematic review include the multi-phase, multi-method, rigorous approach for the identification, screening, and abstraction of CBPs using triangulation of sources across web-based approaches and external, expert reviewers. One method would not have been suitable to capture all CBPs as some programs have dated or incomplete websites. Additionally, we deliberately included terms to capture international DIS programs (e.g., knowledge translation, knowledge exchange). Our processes to check validity and active operations of the programs through thorough website review and individual emails to program contacts strengthened our results.

Although we received responses from just over half of identified programs, the response rate was still higher than the average response rate for online surveys (i.e., 44%) [29]. We were also able to cross-check characteristics abstracted from the website with program self-report data from the survey. Limitations include the possible exclusion of search terms that may be more common in non-English speaking countries or other countries outside of North America and Europe. Given our supplementary search of NIH funding mechanisms, it is also possible that we more easily identified US-based programs. However, we did make substantial efforts to include non-US resources including outreach to DIS experts outside of North America and, when possible, the translation of websites found through the Google searches. We also focused most exclusively on DIS programs; therefore, this list is not an exhaustive reflection of improvement science or quality improvement programs. Moreover, we conducted the searches and reviews in English and are thereby limited to programs with readily translatable webpages. Programs that were recommended by our expert review panel also had a web presence. Programs within larger organizations such as hospitals and research institutes may have DIS capacity building programs not publicly available on websites. We may not have been able to identify programs without a web presence.

## Conclusions

We identified 165 CBPs from 13 countries. This systematic review is the first to comprehensively catalog CBPs around the world and describe their key features and offerings. Our team’s primary next step is to build a searchable online platform featuring each CBP’s descriptive profile to allow DIS researchers and practitioners to collaborate and continually add and update program information. Despite the quantity of DIS programs, several opportunities remain to further enhance and streamline DIS capacity building efforts. Key priorities include sustainment strategies such as advocating for extramural and internal funding to support program infrastructure and capacity building operations rather than relying on traditional research study grants to indirectly support operational activities. There is also a need for formal certification, low-cost, accessible options for learners in LMICs, opportunities for practitioners/non-researchers (e.g., DIS and medical degrees), and opportunities for mid/later stage researchers.

## Supplementary Information

**Additional file 1.** Survey Responses About DIS Product/Resource Expertise^1^.

**Additional file 2. **D&I Science Capacity Building Survey.

**Additional file 3.** PRISMA 2020 Checklist.

## Data Availability

The datasets used during the current study are available from the corresponding author upon request. There are also plans to make the data freely available online in a searchable database.

## Abbreviations

CBP: Capacity building program
DIS: Dissemination and implementation science
NIH: National Institutes of Health
LMIC: Low- and middle-income country
TIDIRC: Training Institute for Dissemination and Implementation Research in Cancer
