# Supplementary material for: A systematic review of dissemination and implementation science capacity building programs around the globe
Source: Implement Sci Commun. 2023 Mar 27;4:34. doi: 10.1186/s43058-023-00405-7 (PMC10041476; doi:10.1186/s43058-023-00405-7)
Supplement: Supplementary file 1 — Additional file 1. Survey Responses About DIS Product/Resource Expertise1. [file 43058_2023_405_MOESM1_ESM.docx]

Additional File 1. Survey Responses About DIS Product/Resource Expertise^1^

| **Program Name** | **Website Link** | **Description of D&I Product/Resource Expertise** |
| --- | --- | --- |
| Behavioral Health Quality Enhancement Research Initiative (QUERI) Program | <https://www.queri.research.va.gov/centers/Behavioral-Health.pdf> | Implementation facilitation - we publish a manual and host a learning hub |
| BRIDGE-C2: Building Research in Implementation & Dissemination to Close Gaps & Achieve Equity in Cancer Control | <https://bridgetoinnovation.org/> | Practice Change Model (Cohen, McDaniel, Crabtree, et al). COVID vaccination toolkits, Implementation Support Strategy Adaptation Tracker, Concise explanation of IS and IS strategies videos. System Science thing that Erin did. |
| CCTSI Dissemination and Implementation Research Core | <https://cctsi.cuanschutz.edu/resources/dissemination> | Designing for Dissemination, Equity, & Sustainability |
| Center for Dissemination and Implementation Science | <https://chicago.medicine.uic.edu/departments/academic-departments/medicine/research/cdis/> | Applications of IS in low to middle income settings; Intervention development and adaptation for new populations; Fidelity assessment; |
| Center for Health Innovation and Implementation Science | [www.hii.iu.edu](http://www.hii.iu.edu) | Agile Implementation Process, Agile Innovation Process, Agile Diffusion Process, Agile Nudge Design, Agile Analytics, Agile Mindset, and Agile Network Engineering |
| Center for Healthcare Delivery Science for Health Equity | <https://sc-ctsi.org/training-education/introduction-to-healthcare-delivery-science> | Healthcare Delivery Science Course |
| Centre for Implementation Science | <https://www.kcl.ac.uk/research/cis> | ImpRes tool and guide (implementation science research development tool)  Implementation outcomes repository (https://implementationoutcomerepository.org) |
| Dissemination and Implementation Launchpad | <https://ictr.wisc.edu/dissemination-implementation-launchpad/> | Design For Dissemination (D4D): https://ictr.wisc.edu/dissemination-implementation-launchpad/di-design-for-dissemination/ Evidence to Implementation (E2I) Dissemination and Implementation Annual Short Course |
| Dissemination and Implementation Methods Unit, NC TraCS Institute | <https://impsci.tracs.unc.edu/> | Theory Comparison and Selection Tool (T-CaST); Community Engaged Dissemination and Implementation (CEDI) Research framework; classification system for implementation strategies; electronic patient reported symptom monitoring tool |
| Dissemination and Implementation Program, Institute of Translational Health Sciences | <https://www.iths.org/community/partners/d-i/> | Promoting the Use of Research Findings in the Real World (Dissemination Workbook): a step-by-step workbook to help translational scientists at any stage in the translational spectrum move their discoveries and interventions into practice  Implementation Planning Guide - an interactive guide and consultation program to help investigators plan and implement health innovations in clinical practice settings  DICE - a web-based tool for tracking the number and types of D&I consultations performed by a D&I team as well as for assessing the outcomes or results of D&I consultations.  Value Champions Training Curriculum/Value Champions Project Workbook - a curriculum for clinical champions on de-implementing low value care. Includes 10 learning modules about essential knowledge and skills and a project workbook to guide a project to reduce the use of a low-value service. |
| Dissemination and Implementation Science Program, ACCORDS, Univ. Colorado SOM | <https://medschool.cuanschutz.edu/accords/cores-and-programs/dissemination-implementation-science-program> | RE-AIM and PRISM conceptual models (not proprietary) Several on-line D&I resources |
| EMPOWER QUERI | <https://www.queri.research.va.gov/centers/EMPOWER.pdf> | rapid qualitative analysis, periodic reflections |
| Equity-focused Dissemination and Implementation Function, University of Rochester CTSI | <https://www.urmc.rochester.edu/clinical-translational-science-institute/clinical-research/equity-focused-dissemination-and-implementation.aspx> | The co-leads contributed to development of the PRIDI (pragmatic, rapid, and iterative dissemination and implementation) framework. |
| Greo | [www.greo.ca](http://www.greo.ca) | Knowledge translation of safer gambling |
| Implementation Science Initiative with Irving Institute CTSA at Columbia University/CUIMC | <https://www.irvinginstitute.columbia.edu/implementation-science> | Curricula for CBPR/implementation science course several you tube channels with content on community-engaged implementation science: https://www.youtube.com/playlist?list=PLvTuZ00BTvIZiW7oqXeBFNF8NXHeFFsds https://www.youtube.com/channel/UCJhGTpULmVIENeYHPDy-jLg/videos monthly seminar series on health equity & imp sci; consultations open to community on imp sci |
| Improvement Science theme of the Yorkshire and Humber Applied Research Collaboration | <https://www.arc-yh.nihr.ac.uk/> | Improvement Science Snapshots that are short, pre-recorded videos covering improvement science methods and approaches. Various research outputs. |
| Institue for Integration of Medicine & Science Improvement Science Research Network | <https://iims.uthscsa.edu/community/resources.html> | Stevens Star Model of Knowledge Transformation; National consensus on EBP competencies; Evidence-Based Practice Inventory |
| Institute for Clinical and Translational Science Engagement, Integration, and Implementation Core | <https://icts.uiowa.edu/investigators/engagement-integration-and-implementation-eii-core> | Iowa Model for Evidence-based Practice |
| Institute for Healthcare Improvement | [www.ihi.org](http://www.ihi.org) | Those at the intersection of improvement sciences and implementation. This includes written tools and frameworks, trainings (online and in person), conferences |
| Interdisciplinary Research in Health Sciences (IRIHS) research unit | <https://www.phc.ox.ac.uk/research/interdisciplinary-research-in-health-sciences> | NASSS framework for studying non-adoption, abandonment and challenges to scale-up, spread and sustainability of digital innovations |
| Intermountain Healthcare Delivery Institute | <https://intermountainhealthcare.org/about/transforming-healthcare/hdi/> | A focus on clinical implementation science shop.The team is comprised of clinicians, applied health services researchers, health system engineers and biomedical informaticists. |
| JBI Evidence Implementation Program | <https://jbi.global/education/evidence-implementation-training> | Conceptual model of EBHC: <https://jbi.global/jbi-approach-to-EBHC>  Software to guide and facilitate implementation planning, context evaluation, implementation, sustainability of practice change  Handbooks for implementation |
| Knowledge Translation and Implementation Coordinating Group at Campbell Collaboration | <https://www.linkedin.com/groups/3746081/> | Systematic reviews |
| Knowledge Translation Program | <https://knowledgetranslation.net/> | Scoping review tools  How to choose your review type (knowledge synthesis) resources for rapid reviews  Many other tools - screening guidelines, organizational readiness for change, decision support tools, more |
| Military Suicide Research Consortium Dissemination and Implementation Core (Core D) | <https://msrc.fsu.edu/about-msrc/dissemination-and-implementation> | In collaboration with our DoD partners, we have developed models for articulating readiness for dissemination and for implementation of evidence-based suicide prevention interventions and clinical research funded by the consortium that could be used in other contexts. |
| National Implementation Research Network | <https://nirn.fpg.unc.edu/> | NIRN primarily uses the Active Implementation Frameworks in our work. The NIRN online Active Implementation Hub contains all products associated with this work. The AI Hub can be located at: https://nirn.fpg.unc.edu/ai-hub. Here you will find virtual learning opportunities around the five active implementation frameworks (Usable Innovations, Stages, Cycles, Teams, Drivers). The most well-known tool located on the site is the Hexagon Tool. This particular tool is used during the innovation selection process in the Exploration Stage. |
| National Institute on Aging Imbedded Pragmatic Alzheimer's disease and AD-Related Dementias Clinical Trials (NIA IMPACT) Collaboratory Implementation Core | <https://impactcollaboratory.org/implementation-core/> | Tip sheets Grand Rounds presentations Various peer reviewed publications Other resources |
| NCI Implementation Science Team | <https://cancercontrol.cancer.gov/is> | TIDIRC training program, IS at a glance, Evidence-Based Cancer Control Programs repository, annual conferences, webinars |
| Nigeria Implementation Science Alliance | http://[nisaresearch.org](http://nisaresearch.org) | Healthy Beginning Initiative (Baby Shower), a congregational platform for health education and intervention |
| Penn Collaborative for CBT and Implementation Science | <https://www.med.upenn.edu/penncollaborative/> | (1)the Beck Community Initiative process and model for implementation, which includes implementation readiness, training and consultation, and sustainability tools (2) LyssnCBT - an artificial intelligence-based tool that supports clinical supervision and automatically measures competence in CBT, Motivational Interviewing, and general clinical skills (3) a web-based training in cognitive behavioral therapy that has been shown to be effective in training providers to competently deliver CBT, using 7% of the resources of a live training |
| Penn Implementation Science Center (PISCE@LDI) | <https://ldi.upenn.edu/about-us/partners/penn-ldi-affiliated-centers/penn-implementation-science-center/> | Subway tool - Lane-Fall, Curran, and Beidas, 2019 |
| PRISE Center (Partnerships for Research in Implementation Science for Equity) | <https://prise.ucsf.edu> | Implementation Science Certificate Program  The Online Certificate in Implementation Science is a part-time program focused on theories and methods relevant to designing strategies to facilitate the uptake of health interventions. Scholars can choose to take individual courses or complete four required and two elective courses to obtain the certificate. The courses and certificate program are designed for people from a broad range of professional settings, both in the U.S. and globally, who are currently engaged in the development, implementation and evaluation of strategies to promote and improve health and health care quality. The target audience includes clinical and public health researchers, quality improvement officers, community-based clinic staff, public health and public policy practitioners and clinicians. |
| Quality Enhancement Research Initiative (QUERI) National Program | <https://www.queri.research.va.gov/> | QUERI trains investigators, staff, and leaders in implementation strategy use; we also have an expansive DEI program (Advancing Diversity in Implementation Leadership) which specifically promotes leadership opportunities in D&I for under-represented groups |
| QUERI Center for Evaluation and Implementation Resources (CEIR) | <https://www.queri.research.va.gov/ceir/default.cfm> | QUERI Implementation Roadmap; VA QUERI Program Evaluation Guide |
| Society for Implementation Research Collaboration | <https://societyforimplementationresearchcollaboration.org> | The Society for Implementation Research Collaboration has several "Networks of Expertise" (e.g., Established Investigators, New Investigators, Practitioners, Students, etc.) that provide mentorship and collaboration opportunities. Additionally, SIRC has supported specific initiatives to advance specific areas of implementation science and practice (e.g., initiatives to advance measurement, the study of mechanisms in implementation science, and the infrastructure needed to advance implementation practice and research). |
| The Center for Implementation | <https://thecenterforimplementation.com/> | Tools (Map2Adapt), infographics (e.g., for power and trust), frameworks (e.g., StrategEase). These are embedded in tools, training, and technical assistance that we provide to professionals and researchers who are implementing or supporting implementation. |
| The Impact Center at FPG | <https://impact.fpg.unc.edu/> | (1) Integrated Theory of Change for the Successful, Sustainable Scale-up of Evidence-Based Interventions (Aldridge et al., 2018; National Academies of Sciences, Engineering, and Medicine, 2019); (2) Practice Model for External Implementation Support, including principles, core practice components, practice activities, practice outcomes, and related logic model (Aldridge et al., 2022a & 2022b - manuscripts in preparation); (3) Community Capacity Assessment (Aldridge et al., 2016); (4) Implementation Drivers Assessment (Aldridge et al., 2016); (5) Local-Regulation of Implementation Processes Scale (Roppolo & Aldridge, 2019) |
| The National Collaborating Centre for Methods and Tools | <https://www.nccmt.ca/> | "Learn-as-you-go" (LAGO) study designs |
| The University of Washington Department of Global Health Implementation Science Program | [ImpSciUW.org](http://ImpSciUW.org) | Systems Analysis and Improvement Approach (SAIA), an implementation strategy developed by Kenneth Sherr. FIM, AIM, and IAM measures of implementation feasibility, acceptability, and appropriateness, developed by Bryan Weiner. |
| UAMS Center for Implementation Research | <https://tri.uams.edu/resources-and-services/tri-services/the-center-for-implementation-research/> | Hybrid designs, Evidence-Based Quality Improvement |
| UC San Diego ACTRI DISC | <https://medschool.ucsd.edu/research/actri/centers/DIR/Pages/default.aspx> | RE-AIM EPIS PRISM D&I Models website |
| UCSF Implementation Science Training Program | <https://epibiostat.ucsf.edu/online-certificate-implementation-science> | Introduction to Implementation Science Theory and Design Community-Engaged Research Human-Centered Design Program Evaluation in Clinical and Public Health Settings Designing Individual-Level Implementation Strategies Qualitative and Mixed Methods Research Study Designs for Intervention Research in Real-World Settings Designing Interventions to Change Organizational Behavior Translating Evidence into Policy |
| UK Implementation Society | <https://www.ukimplementation.org.uk/> | Returning aggregate study findings to research participants through different methods and deliverables. |
| University of Utah Clinical and Translational Sciences Institute | <https://ctsi.utah.edu/> | Implementation Research Logic Model (IRLM)(Smith, Li, & Rafferty, 2020) and the Longitudinal Implementation Strategies Tracking System (LISTS) (Smith et al. 2022) |
| UTHealth Institute for Implementation Science | in development | Implementation Mapping, Intervention Mapping, Adaptation including IM ADAPT Online tool for finding and adapting EBIs, geospatial modeling, ECHO hub, |
| Vanderbilt Institute for Clinical and Translational Research - Dissemination Core | <https://victr.vumc.org/dissemination-of-research-results/> | Online learning modules, educational videos, webinars, guidebooks, Knowledge Broker mentoring program, workshops, online resources, online repositories |

^1^ These responses were directly abstracted from the survey.
