# Supplementary material for: A systematic review of dissemination and implementation science capacity building programs around the globe
Source: Implement Sci Commun. 2023 Mar 27;4:34. doi: 10.1186/s43058-023-00405-7 (PMC10041476; doi:10.1186/s43058-023-00405-7)
Supplement: Supplementary file 2 — Additional file 2. D&I Science Capacity Building Survey. [file 43058_2023_405_MOESM2_ESM.pdf]

D&I Science Capacity Building Survey

Start of Block: Block 6

Capacity Building in
Dissemination and Implementation Science (D&I) You are being invited to participate in a project titled “Capacity Building in Dissemination and Implementation Science (D&I)”. This study is being led by Borsika Rabin and Nicole Stadnick from the University of California San Diego Altman Clinical and Translational Research Institute Dissemination and Implementation Science Center (UC San Diego ACTRI DISC) to learn more about D&I capacity building initiatives nationally and internationally. You were selected to participate because you have been identified online or by colleagues as the primary contact for your D&I science center or program. Data from this project will be used to describe your center or program in publications and databases to share with the larger research and practice community.
We use the term dissemination and implementation to describe activities to advance the dissemination, adoption, implementation, and sustained use of evidence (i.e., program, procedure, policy, practice, pill, principle, product) in various settings including community or clinical settings. We realize that different countries use different terms to describe these activities including but not limited to knowledge translation, knowledge exchange, scale-up, etc. Our objective is to include all of these broadly defined programs and centers so that all are included in our database.
This survey should take 20-30 minutes to complete. Your participation in this survey is entirely voluntary and you can withdraw at any time by simply exiting. All data collected up to the point of exiting the survey will be retained. If you wish to have all of your data removed, please contact Clare Viglione at cviglione@health.ucsd.edu. You are also free to skip any question that you choose. Choosing not to participate or withdrawing will result in no penalty or loss of benefits to which you are entitled. If you want additional information or have questions or research-related problems, you may reach Dr. Borsika Rabin at barabin@health.ucsd.edu.
By clicking “I agree” you are indicating that you have read this consent form and agree to participate in this survey. Click here **to print a copy of this page for your records.**

○ I Agree

○ I Do Not Agree

Skip To: End of Survey If Capacity Building in Dissemination and Implementation Science (D&I) You are being invited to p... = I Do Not Agree

End of Block: Block 6

Start of Block: Background

Thank you for agreeing to complete this survey! We will be asking questions about your Dissemination and Implementation (D&I) Science Program(s). As you are filling out the form, it may be helpful to get input from your colleagues to answer questions and provide the most up-to-date and comprehensive information. This survey is expected to take 20-30 minutes to complete.

Please note, this survey data will be used to create an inventory of D&I programs and centers and to describe their general characteristics and activities.

End of Block: Background

Start of Block: Block 9

Q1 Enter the name of your D&I center or program:*

____

Q2 Is the center or program affiliated with an academic institution?

- ◯ Yes
- ◯ No

Display This Question:

If Is the center or program affiliated with an academic institution? = No

If the center or program is associated with an organization, please enter the name(s):

____

Display This Question:

If Is the center or program affiliated with an academic institution? = Yes

Please enter the academic institution(s):

____

Display This Question:

If Is the center or program affiliated with an academic institution? = Yes

Within which department or division is the primary center/program housed? (Enter N/A, if none)

____

Display This Question:

If Is the center or program affiliated with an academic institution? = Yes

Name of secondary department or division? (Enter N/A, if none)

____

*

Q3 What year was the center or program established?

____

End of Block: Block 9

Start of Block: Center/Program Primary Contact information

Q4 Who is the primary contact for your center or program? Please note, we will use this email address to follow up and share results of the study and discuss the database.

First Name*

____

Q5 Last Name*

____

Q6 Professional Title (as it relates to the Center or Program)*

____

Q7 Email*

____

Q8 Please indicate if there are any other director(s) or leader(s) in your program/center? (if different from primary contact)

- ◯ Last Name, First Name ____
- ◯ Last Name, First Name ____
- ◯ Last Name, First Name ____
- ◯ Last Name, First Name ____

Q9 Is your center/program located in the US?

- ◯ Yes
- ◯ No

Display This Question:

If Is your center/program located in the US? = Yes

State*

▼ Alabama,AL ... Northern Mariana Islands

Display This Question:

If Is your center/program located in the US? = Yes

City*

____

Display This Question:

If Is your center/program located in the US? = No

Country*

____

Q10 Center/program website and social media (Select all that apply and fill in profile name or URL)

- ▢ Webpage: ____
- ▢ Twitter handle: ____
- ▢ Facebook page: ____
- ▢ LinkedIn account: ____
- ▢ Other platform: ____

End of Block: Center/Program Primary Contact information

Start of Block: Member characteristics (number, type, background of members)

Q11 Does your center or program keep track of "members"? By "member" we mean someone who is affiliated with the center, receives updates and announcements, and participates in one or more program activities.

- Yes
- No

Display This Question:

If Does your center or program keep track of "members"? By "member" we mean someone who is affiliate... = Yes

How many members are part of your center/program?

- 0-25
- 26-50
- 51-75
- 75-100
- 101-150
- 151-200
- 201-250
- >250
- Not sure

Q12 How many faculty and staff team members hold a formal position (i.e., a paid and/or named role) as part of this center/program? Please insert number of positions for each category.

- ☐ Faculty ____
- ☐ Staff ____

End of Block: Member characteristics (number, type, background of members)

Start of Block: Block 8

Q15 What populations does your center/program serve? (select all that apply)

- [ ] Pediatrics
- [ ] Adults
- [ ] Older Adults
- [ ] Women
- [ ] LGBTQ+
- [ ] Indigenous communities
- [ ] Rural
- [ ] Urban
- [ ] Clinical
- [ ] Houseless/Homeless
- [ ] General community
- [ ] Specific ethnic/racial group (please specify) ________________
- [ ] Underserved/marginalized communities (please specifiy) ________________
- [ ] Other (please describe) ________________
- [ ] ⊗ We don’t serve a specific population.

Q16 Describe your D&I center with 5 key words (e.g., mixed methods, lifecourse, translational, etc.)

- ○ Word 1 ____
- ○ Word 2 ____
- ○ Word 3 ____
- ○ Word 4 ____
- ○ Word 5 ____

End of Block: Block 8

Start of Block: D&I product

Q17 Does your D&I center or program collaborate or engage with community partners?

- ○ Yes
- ○ No
- ○ Unsure

Display This Question:

If Does your D&I center or program collaborate or engage with community partners? = Yes

Q57 Please describe the types of community partners your D&I center or program works with:

____

____

____

____

____

Q18 Are there unique D&I products or resources that your center has specific expertise in (e.g., a proprietary model, method, framework, or instrument/measure)?

◯ Yes

◯ No

Display This Question:

If Are there unique D&I products or resources that your center has specific expertise in (e.g., a pr... = Yes

Please describe the D&I product(s):

____

____

____

____

____

End of Block: D&I product

Start of Block: Inputs (Financial and in-kind resources)

Q19 What are the sources of financial support for the Center/Program?

- ☐ Internal institutional funds (School of Medicine, School of Public Health, etc.)
- ☐ CTSA (Clinical and Translational Science Awards) funding
- ☐ Internal department funds
- ☐ Research grants, center/program grants
- ☐ Funding from non-profits
- ☐ Other ____

End of Block: Inputs (Financial and in-kind resources)

Start of Block: Activities

Q20 What Dissemination and Implementation methodologies is your center/program specialized in? (select all that apply)

- ☐ D&I models/frameworks
- ☐ Adaptations
- ☐ Fidelity
- ☐ Implementation strategies
- ☐ Quantitative methods
- ☐ Qualitative methods
- ☐ Mixed methods
- ☐ D&I measures
- ☐ Sustainability/Sustainment
- ☐ Organizational science
- ☐ Team science
- ☐ Scale up
- ☐ Scale out
- ☐ Stakeholder engagement and partnerships
- ☐ Study designs
- ☐ Cost analysis

- [ ] Designing for dissemination and implementation
- [ ] Other (please describe) ____

Q21 Are your funding sources primarily short-term investments (start-up funds, project-specific funds) or long-term/ongoing funding? Please describe.

____

____

____

____

____

Q22 What services/activities does your center/program offer? (select all that apply)

- ☐ D&I Training & Education
- ☐ D&I Mentorship
- ☐ D&I Internship Academic for Students/Trainees
- ☐ D&I Consultation
- ☐ D&I Professional Networking Opportunities
- ☐ D&I Grant Development Support
- ☐ D&I Technical Assistance
- ☐ D&I Resources and Tools
- ☐ D&I Other specialized areas ____

Display This Question:

If What services/activities does your center/program offer? (select all that apply) = D&I Training & Education

What types of training and/or education does your center/program offer? "Program" indicates structured curriculum with at least 3 D&I courses. (select all that apply)

- ☐ Webinars/Seminars
- ☐ Bootcamps
- ☐ Workshops
- ☐ Undergraduate course
- ☐ Undergraduate D&I Program
- ☐ Graduate Course
- ☐ Graduate D&I Program (Doctoral)
- ☐ Graduate D&I Program (Masters)
- ☐ Graduate D&I Program (Certificate)
- ☐ Master of Public Health program
- ☐ Public Health PhD programs
- ☐ Integrated training in MD/RN
- ☐ Invited Guest Speakers
- ☐ Other (please describe)

____

Display This Question:

If What services/activities does your center/program offer? (select all that apply) = D&I Mentorship

What types of D&I mentorship does your center/program offer? (select all that apply)

- ☐ Positions for official (grant-sponsored) trainees
- ☐ Positions for faculty/staff mentorship
- ☐ Positions for student mentorship
- ☐ Internships
- ☐ Consultations
- ☐ Other (please describe) ________________

Display This Question:

If What services/activities does your center/program offer? (select all that apply) = D&I Consultation

What types of D&I consultation does your center/program offer? (select all that apply)

- ☐ Individual (1-1) consultation
- ☐ Small group consultation
- ☐ Other (please describe) ________________

Display This Question:

If What types of D&I consultation does your center/program offer? (select all that apply) = Individual (1-1) consultation

Or What types of D&I consultation does your center/program offer? (select all that apply) = Small group consultation

Or What types of D&I consultation does your center/program offer? (select all that apply) = Other (please describe)

Do you require payment for D&I consultation services?

- ○ Yes
- ○ No
- ○ Depends/situatlional

Display This Question:

If What services/activities does your center/program offer? (select all that apply) = D&I Professional Networking Opportunities

What types of D&I Professional Networking opportunities does your center/program offer? (select all that apply)

- ▢ Professional networking conferences
- ▢ Journal Club style meetings
- ▢ Virtual/In-person networking events
- ▢ Informal gatherings/meet-ups
- ▢ Other (please describe) ________________

Display This Question:

If What services/activities does your center/program offer? (select all that apply) = D&I Grant Development Support

What types of D&I Grant Development does your center/program offer? (select all that apply)

- ☐ Recorded video instructions
- ☐ In-person/Web-based trainings
- ☐ Works in Progress meetings
- ☐ Working groups
- ☐ Other (please describe) ________________

Display This Question:

If What services/activities does your center/program offer? (select all that apply) = D&I Technical Assistance

What types of D&I Technical Assistance does your center/program offer? (select all that apply)

- ☐ Recorded video tutorials/trainings
- ☐ In-person or virtual training (i.e., synchronous meeting)
- ☐ Journal club
- ☐ Working groups
- ☐ Other (please describe) ________________

Display This Question:

If What services/activities does your center/program offer? (select all that apply) = D&I Resources and Tools

What types of D&I Resources and Tools does your center/program offer? (select all that apply)

- [ ] Educational materials
- [ ] Interactive web-based resources
- [ ] Training videos
- [ ] Other (please describe)

____

Q23 How does your center/program measure productivity/outcomes? (Select all that apply)

- [ ] # of Grants
- [ ] # of Publications
- [ ] # of Mentees
- [ ] # of Conferences hosted
- [ ] # of Meetings hosted
- [ ] # of Active Members
- [ ] # of Individuals who receive marketing communications
- [ ] # of New collaborations
- [ ] # of Proposals submitted
- [ ] Member Satisfaction
- [ ] Social media analytics
- [ ] Other (please describe) ____

Q24 How frequently do you evaluate productivity/outcomes?

- ◯ Once a month
- ◯ Every three months
- ◯ Every 6 months
- ◯ Once a year
- ◯ Other (please describe) ____

Q25 Does your center/program use D&I competencies to guide your research and training activities?
D&I competencies might include: defining a process for dissemination/implementation, applying additional D&I strategies within your research model, etc.

- ◯ Yes
- ◯ No
- ◯ Sometimes
- ◯ Not sure

Display This Question:

If Does your center/program use D&I competencies to guide your research and training activities? D&... = Yes

What competencies do you use? Please describe.

D&I competencies might include: defining a process for dissemination/implementation, applying additional D&I strategies within your research model, etc.

____

____

____

____

____

Q26 Is your center or program using an evaluation framework to guide evaluation of your center training program activities (e.g., EPIS, RE-AIM, WUNDIR Logic Model, Translational Science Benefits Model, etc.)? Please describe.

____

____

____

____

____

Q27 Does your center/program evaluate any of the following? Please select all that apply

- ☐ Member satisfaction
- ☐ Member engagement
- ☐ D&I knowledge
- ☐ Productivity (e.g. # grants, # publications)
- ☐ D&I skills
- ☐ Training effectiveness
- ☐ Other ____

Q28 Does your center/program use [Translational Science Benefits Indicators] to evaluate any of your research and training activities? These encompass downstream public health impacts such as changes to healthcare services, healthcare cost-effectiveness, or policy-level outcomes.

- ◯ Yes
- ◯ No
- ◯ Not sure

Display This Question:

If Does your center/program use Translational Science Benefits Indicators to evaluate any of your re... = Yes

Q64 Which Translational Science Benefits Indicators categories do you use for evaluation? Select all that apply.

- ▢ Clinical & Medical (e.g., biomedical technology, drugs, diagnostic guidelines)
- ▢ Community & Public Health (e.g., health care delivery, accessibility, life expectancy)
- ▢ Economic (e.g., cost effectiveness, cost savings, societal cost of illness)
- ▢ Policy & Legislative (e.g., committee participation, policies, expert testimony)

Q29 Would you be willing to participate in a 60-minute follow-up discussion about your center/program via Zoom?

- ◯ Yes
- ◯ No

Q30 Are you affiliated or familiar with any other D&I center(s)/program(s)?

◯ Yes

◯ No

Display This Question:

If Are you affiliated or familiar with any other D&I center(s)/program(s)? = Yes

Q63 List any other D&I center(s)/program(s) that you are affiliated or familiar with. Please separate centers by using a semicolon (;)

____

____

____

____

____

Thank you for your time!

Please don't hesitate to contact us at disc@health.ucsd.edu.

End of Block: Activities

Start of Block: Types of D&I Research and Topics
